# Current species protection does not serve its porpoise—Knowledge gaps on the impact of pressures on the Critically Endangered Baltic Proper harbour porpoise population, and future recommendations for its protection

**DOI:** 10.1002/ece3.70156

**Published:** 2024-09-12

**Authors:** Sven Koschinski, Kylie Owen, Kristina Lehnert, Katarzyna Kamińska

**Affiliations:** ^1^ Meereszoologie Nehmten Germany; ^2^ Department of Population Analysis and Monitoring Swedish Museum of Natural History Stockholm Sweden; ^3^ Institute for Terrestrial and Aquatic Wildlife Research University of Veterinary Medicine Hannover Hannover Germany; ^4^ Department of Fisheries Ministry of Agriculture and Rural Development Warsaw Poland

**Keywords:** Baltic, biology, cetacean, conservation, ecology, threats

## Abstract

Successful management requires information on pressures that threaten a species and areas where conservation actions are needed. The Baltic Proper harbour porpoise population was first listed as Critically Endangered by the International Union for the Conservation of Nature in 2008. Now, 16 years later, there is no change in conservation status despite ample conservation policy calling for its protection and an urgent need for management action to protect this population. Here, we provide an overview of the current status of the population, highlight knowledge gaps on the impact of pressures, and make recommendations for management of anthropogenic activities. Based on an exceeded limit for anthropogenic mortality, the high concentrations of contaminants in the Baltic Sea, combined with reductions in prey availability and increases in underwater noise, it is inferred that this population is likely still decreasing in size and conservation action becomes more urgent. As bycatch and unprotected underwater explosions result in direct mortality, they must be reduced to zero. Inputs of contaminants, waste, and existing and emerging noise sources should be minimised and regulated. Additionally, ecosystem‐based sustainable management of fisheries is paramount in order to ensure prey availability, and maintain a healthy Baltic Sea. Stranding networks to routinely assess individuals for genetic population assignment and health need to be expanded, to identify rare samples from this population. Knowledge is still scarce on the population‐level impact of each threat, along with the cumulative impact of multiple pressures on the population. However, the current knowledge and management instruments are sufficient to apply effective protection for the population now. While bycatch is the main pressure impacting this population, urgent conservation action is needed across all anthropogenic activities. Extinction of the Baltic Proper harbour porpoise population is a choice: decision‐makers have the fate of this genetically and biologically distinct marine mammal population in their hands.

## Graphical abstract

The Critically Endangered Baltic Proper harbour porpoise population is likely still decreasing in size due to an exceeded limit for anthropogenic mortality, the high concentrations of contaminants in the Baltic Sea, reductions in prey availability, and increases in underwater noise. As bycatch and unprotected underwater explosions result in direct mortality of harbour porpoises, they must be reduced to zero, with further management action also required across all sectors to reduce the impact of other pressures. Extinction of the Baltic Proper harbour porpoise population is a choice: the management instruments available are sufficient to apply effective protection for the population now and decision makers have the fate of this genetically and biologically distinct marine mammal population in their hands.

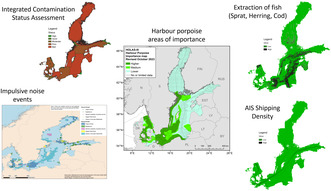

## 1 INTRODUCTION

### 1.1 The Baltic proper harbour porpoise population

**FIGURE 1:**
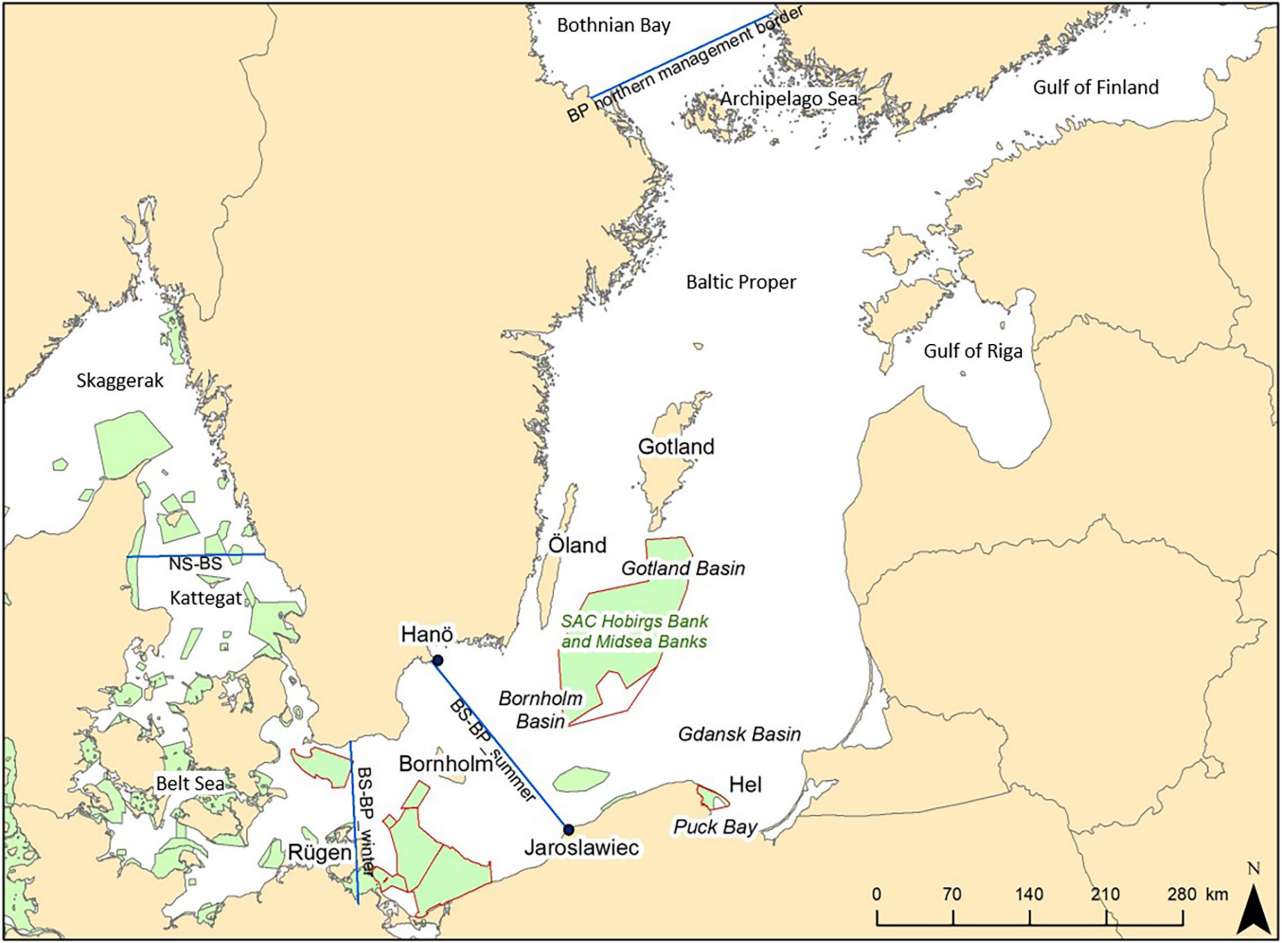
Map of the management borders for all three‐harbour porpoise (*Phocoena phocoena*) populations in the Baltic region (North Sea population (NS), Belt Sea population (BS) and the Baltic Proper population (BP)) are shown. The Natura 2000 sites where harbour porpoises are listed are shown in green. Sites outlined in red are those to which seasonal or year‐round closures for fisheries apply.

Successful management of anthropogenic activities with respect to species conservation relies on information on the current status of populations and the impact of threats. In the Baltic Sea region, three populations of harbour porpoises (*Phocoena phocoena* (Linnaeus, 1758)) are recognised: The North Sea population, the Belt Sea population and the Baltic Proper population (Figure 1). The Baltic Proper population is listed as Critically Endangered (CR) by the Baltic Marine Environment Protection Commission (HELCOM) and the International Union for the Conservation of Nature (IUCN) (Carlström et al., 2023; HELCOM, 2013). Genetic and morphometric studies have concluded that the Baltic Proper harbour porpoise forms a separate population distinct from those living in the Belt Seas and the Kattegat and with a further distinction to the population of the Skagerrak and the North Sea (e.g. Autenrieth et al., 2024; Celemín et al., 2023; Galatius et al., 2012; Huggenberger et al., 2002; Lah et al., 2016; Wiemann et al., 2010). This is also supported by studies using acoustic monitoring and satellite tracking that describe seasonal migrations and spatial separation during the breeding season from the neighbouring Belt Sea population (Carlén et al., 2018; Sveegaard et al., 2015).

### 1.2 Legal structures enacted with the aim of conserving harbour porpoises

All harbour porpoise populations in the European Union (EU) region are protected under the EU Habitats Directive (HD), with the species listed in Annex IV, which legally requires Member States to establish a system of strict protection. Member States must report on population status, range, habitat and future prospects (Article 17, in relation to Favourable Reference Values (FRVs)) every 6 years. For three consecutive assessment periods, all relevant EU Member State assessments and the EU biogeographical assessment have classified the conservation status of the Baltic Proper Harbour porpoise as ‘Unfavourable‐Bad’ (U2). The species is also listed in Annex II of the HD, requiring the designation of special areas of conservation (SACs) in a coherent Natura 2000 network. The Swedish SAC *Hoburgs Bank and Midsea Banks* (SE0330308) covers an area of year‐round importance for the population, likely including parts of an important breeding ground (Carlén et al., 2018) (Figure 1). Other protected areas of seasonal importance are also shown in Figure 1.

For many years, for most of the SACs shown in Figure 1, no specific measures to protect harbour porpoises were in place. Unfortunately, there is large variability in the quality and level of detail in management or conservation plans between these sites, and many of the objectives are not SMART (Specific, Measurable, Achievable, Relevant and Time‐Bound) objectives as required by the EU to effectively manage SACs. Management plans for these SACs should include definitions of specific conservation objectives and timeframes, identification of threats and their subsequent pressures, identification of necessary and effective protection measures, as well as provisions for monitoring of the species and the impact of any implemented measures.

The harbour porpoise is further protected under the EU Marine Strategy Framework Directive (MSFD), to maintain biological diversity under Descriptor 1 (D1). Under this Descriptor, Member States are required to establish thresholds for indicators for the species achieving Good Environmental Status (GES) for five criteria (mortality rate D1C1, population abundance D1C2, population demographics D1C3, species distributional range and pattern D1C4, and habitat for the species D1C5). Member States are required to monitor the harbour porpoise, to provide data to assess the indicators and detect changes in species status early. The thresholds for indicators are developed and agreed regionally, within regional conventions such as HELCOM, who also prepare the Holistic Assessments on the state of the Baltic Sea (HOLAS) every 6 years. During the HOLAS 3 assessment in 2022, the Baltic Proper population did not achieve GES for bycatch, abundance or distribution (HELCOM, 2023a, 2023b, 2023c; respectively); the only three indicators assessed for this population. Additionally, the assessment of abundance and distribution was a qualitative assessment based on expert opinion only, as quantitative indicators are lacking.

HELCOM produces the Baltic Sea Action Plan (BSAP) (HELCOM, 2021a), which includes actions to reduce ‘knowledge gaps related to areas of high by‐catch risk’ and by 2028 ‘additional areas of high by‐catch risk for both Baltic Sea populations are to be determined’. Additionally, by 2025 ‘possible mitigation measures for threats other than by‐catch’ are to be identified. The first step of this process is to ‘specify knowledge gaps on all threats to the Baltic Proper harbour porpoise population’; a requirement which led to the need for this study.

The Regulation on the EU Common Fisheries Policy (1380/2013) (CFP) provides protection for harbour porpoises and their prey. It states that the impact of fishing on the marine environment should be minimised, by reducing bycatch of harbour porpoises and ensuring sustainable use of fish species. Under the CFP, Member States put forward Joint Recommendations for the adoption of conservation measures which then are adopted in the form of delegated regulations. In the Baltic Sea, the Member States' are organised in the Baltic Sea Fisheries Forum (BALTFISH). Joint Recommendations are agreed upon by the fisheries directors organised in the BALTFISH high‐level group.

In July 2019, a letter by a number of environmental NGOs was sent to the European Commission calling for Emergency Measures to mitigate bycatch of the Baltic Proper harbour porpoise. Emergency Measures are a component of the CFP (Article 12), as ‘Commission measures in case of a serious threat to marine biological resources’, where the Commission should ‘adopt immediately applicable implementing acts applicable for a maximum period of six months’. This call for emergency measures resulted in the EU requesting the International Council for the Exploration of the Sea (ICES) for Special request advice, to review the current status and threats to the population, and recommend appropriate measures. In May 2020, this Special request advice (ICES, 2020a) was sent to the EU outlining Emergency measures to prevent bycatch of the Baltic Proper population both inside and outside of protected areas. As a result, in December 2021, the Commission Delegated Regulation (EU) 2022/303 entered into force containing provisions for year‐round or seasonal closures for static net fisheries or the use of acoustic deterrent devices (so‐called ‘pingers’) in fishing métiers known to cause the most bycatch of harbour porpoises within protected areas (Figure 1).

In 2020, the EU Commission issued a Letter of Formal Notice to Sweden for not sufficiently implementing the measures required under the HD and the CFP, urging Sweden to take action to reduce bycatch of harbour porpoises. In February 2024, as ‘harbour porpoises are still not protected in Sweden to the requisite legal standard’ the European Commission sent a Reasoned Opinion to Sweden (INFR(2020)4037) for ‘failure to fulfil obligations under the Habitats Directive (Directive 92/43/EEC) with regard to the harbour porpoise’. Sweden had 2 months to respond, and to take the necessary measures. If unsuccessful, the Commission may refer the case to the Court of Justice of the European Union.

There are currently no conservation measures agreed upon outside of protected areas, with the exception of one small area, part of the Middle bank, in Polish waters. Based on the scientific advice (ICES, 2020a), the use of pingers has been proposed as a useful measure to lower the bycatch rate. However, during the ASCOBANS Jastarnia Group meeting in 2021, a few countries informed that according to their militaries, pingers were considered a national security problem, and suggested putting forward options for the conservation of harbour porpoises other than the widespread use of pingers (ASCOBANS, 2021).

In February 2023, the EU Commission released a new Action Plan (European Commission, 2023) for ‘Protecting and restoring marine ecosystems for sustainable and resilient fisheries’, where the Commission calls on Member States to ‘adopt national measures or submit joint recommendations to the Commission to minimise by‐catch (or reduce it to the level that enables the full recovery of the populations) of harbour porpoise in the Baltic Proper’ by the end of 2023. For the Baltic Proper population, this is zero bycatch (HELCOM, 2023a; NAMMCO & IMR, 2019). To the best of our knowledge, this action plan did not result in any implementation of policy protecting Baltic Proper harbour porpoises by the end of 2023. However, the EU Commission has requested ICES for more advice on how to develop a proxy for the risk of bycatch of Baltic Proper harbour porpoise, based on the fishing gears most likely to cause bycatch and assessment of fishing effort in the region. This advice is expected to be available by the end of 2024.

Harbour porpoises are protected under international conventions such as the Convention on the Conservation of European Wildlife and Natural Habitats (Bern Convention), the Convention on Biological Diversity (CBD), the Convention on the Conservation of Migratory Species of Wild Animals (CMS, also known as Bonn Convention) with their Agreement on the Conservation of Small Cetaceans of the Baltic, North East Atlantic, Irish and North Seas (ASCOBANS). In 2024, the Baltic Proper harbour porpoise population was added to the species list in Appendix I of CMS, which indicates to Parties that the species that should be provided immeditate protection.

The conservation of harbour porpoises is the centre of regionally agreed recommendations such as the ASCOBANS Recovery Plan for Baltic Harbour Porpoises (Jastarnia Plan) (ASCOBANS, 2016), HELCOM Recommendation 17/2 (HELCOM, 2020) and HELCOM Recommendation 37/2 (HELCOM, 2016). Additionally, the MSFD Descriptor 11 on ‘Energy and Noise’ states that both impulsive and continuous low‐frequency sound sources ‘should not exceed levels that adversely affect populations of marine animals’. Similarly, the MSFD Descriptor 8 on ‘Contaminants’ highlight the ‘need to look at health of species regarding contaminant effects in European waters’. In conclusion, there are multiple legal frameworks and much action on paper aimed at protecting the Critically Endangered Baltic Proper harbour porpoise.

### 1.3 Aims

The aim of this study was to provide an overview of the current state of knowledge on the Baltic Proper harbour porpoise, including information on biology and ecology required for assessment of the status of the population under the legal structures described above. We then summarise what is known about the impact of pressures from various threats, and identify knowledge gaps. Conclusions and recommendations for management actions which will provide the most benefit to the conservation of the Baltic proper harbour porpoise based on a classification of pressures involved are given.

## 2 CURRENT KNOWLEDGE ON THE BALTIC PROPER HARBOUR PORPOISE POPULATION

In the frame of the HD and MSFD (Section 1.2), EU Member States are obliged to monitor, assess and report on the status of harbour porpoises and take measures to achieve Favourable Conservation Status (FCS) (under the HD) or GES (under the MSFD). Assessments under both Directives are based on partly overlapping categories: mortality, abundance, demography, distribution and habitat in the MSFD and population, range, habitat and future prospects in the HD. We summarise how these categories are currently assessed, and what information is available for assessment of the Baltic Proper harbour porpoise in each reporting round.

### 2.1 Mortality rate

In the frame of the MSFD assessment, HELCOM developed an indicator on the *Number of drowned mammals and waterbirds in fishing gear* (HELCOM, 2023a). Static nets such as gillnets and trammel nets account for most harbour porpoise bycatch (Section 3.1.1). However, specific knowledge of the Baltic Proper population is scarce. The bycatch indicator and its thresholds acknowledge that besides bycatch there are other anthropogenic causes for mortality, such as underwater explosions which can cause direct mortality (Siebert et al., 2022). Further, sub‐lethal impacts of noise can lead to compromised fitness (e.g. reduced reproductive potential and survival rate associated with disturbance, habitat alteration, induced for example by overfishing or coastal development and accumulation of pollutants) and add to anthropogenic mortality (Lamoni, 2023; Schaffeld et al., 2020; Scotti et al., 2022; Sonne et al., 2020).

While a threshold for zero mortality has been regionally agreed (HELCOM, 2023a), data on the mortality rate of this population are scarce. This lack of knowledge is due to poor implementation of fisheries monitoring programmes to observe bycatch. The reluctant reporting of bycaught porpoises and low‐population size minimises the chance that stranded animals are found or submitted for post‐mortem analyses to determine the cause of death (IJsseldijk et al., 2021).

### 2.2 Population abundance

The HELCOM indicator *Abundance and population trends of harbour porpoises* (HELCOM, 2023b) evaluates whether the absolute abundance and the trend in abundance of harbour porpoises in the Baltic Proper is adversely affected due to anthropogenic pressure and whether its long‐term viability is ensured.

Although there are no reliable estimates of pre‐exploitation population size, historical data from bounty schemes, bycatch records and observations of dead stranded animals show that the species was numerous in the Baltic Proper, and in Bothnian Bay, during the first half of the 1900s (Johansen, 1929; Lönnberg, 1940; Psuty, 2013; Tägström, 1940). Additionally, based on high bycatch numbers (~50 animals in 12 months), it still appears to have been relatively abundant in an area stretching from Hanö Bight to the waters surrounding Gotland in the early 1960s (Figure 1) (Lindroth, 1962).

Based on acoustic data collected during the SAMBAH project (https://www.sambah.org/), in 2011–2013 the abundance of the Baltic Proper harbour porpoise was estimated to be 491 individuals (CV = 0.68; 95% confidence interval = 71–1105) and is the only abundance estimate to date (Amundin et al., 2022). Based on a dynamic production model, population abundance and annual bycatch estimates, the population was estimated to have declined by 9% from 2009 to 2017 (NAMMCO & IMR, 2019). However, population‐wide trend data are not possible at this stage, due to a lack of repeated surveys of population abundance.

Results of national acoustic monitoring studies show varying results, and cannot provide information on population abundance. At a limited number of stations in Danish, Polish and Swedish waters, detection rates increased compared to SAMBAH results in recent years (Owen et al., 2021; Sveegaard, 2020; Swistun et al., 2019). In contrast, a towed acoustic survey in the Natura 2000 site *Hoburgs Bank and Midsea Banks* (Figure 1) resulted in similar densities of harbour porpoises within the Natura 2000 area as during the SAMBAH survey (Boisseau et al., 2022). However, due to differences in the methods used, the limited area monitored, and a lack of supplementary data required on detection probability, it remains unknown whether the increased detection rates in some areas are a result of changes in population abundance or shifts in distribution over time.

Regularly updated estimates of population abundance (i.e. through repeated SAMBAH surveys, such as SAMBAH II planned from 2024 to 2025) are needed in order to accurately inform management on the impact of any implemented conservation action. Additionally, an estimate of the historical population size, potentially using genetics (Celemín et al., 2023), is needed in order to set quantitative indicator thresholds for species assessments of abundance.

### 2.3 Population demographics

There is currently no defined indicator on the population demographics of the Baltic Proper harbour porpoise population. Historic data on population demographics are lacking as there was no scientific interest in this species when the population was abundant, and putative population boundaries were not defined until recently. There is also a lack of recent samples available due to the low‐population size, meaning that no population‐specific information on life history parameters and age structure is available. This situation is unlikely to change in the near future until the population begins to recover. Thus, the information provided here is derived mostly from the neighbouring Belt Sea population, which is very similar from a biological perspective. However, there may be differences in life history parameters between the two populations or in the life history parameters of a population over time.

Female harbour porpoises give birth to one calf after a gestation period of 10–11 months (ASCOBANS, 2016; Börjesson & Read, 2003; Hasselmeier et al., 2004; Lockyer & Kinze, 2003). In German Baltic Sea waters, most births are recorded between June and August, however, differences between the two populations in German waters are possible (Börjesson & Read, 2003; Hasselmeier et al., 2004). Mating takes place shortly after birth. Testes with full spermatogenic activity in the peak breeding season were found to occur in late June and July in male porpoises in the North‐ and Baltic Sea (Kesselring et al., 2019). In the area with the highest detection rates for the Baltic Proper population around the Midsea Banks, bimodal peaks in detection rates have been observed (Owen et al., 2021). These peaks have been hypothesised to give potential insight into the breeding behaviour of the population, with the first peak (May) potentially coinciding with calving, and the latter (September/October) with the arrival of males; although this is late compared to neighbouring populations (Owen et al., 2021). Lactation is for about 8–9 months (Sørensen & Kinze, 1994). Juveniles begin to forage for their own food from the age of 5–6 months. Females and offspring usually remain together until the calf begins to forage independently at around 11 months of age (Tielmann et al., 2007) and/or until the birth of the next offspring (Schulze, 1996).

In a long‐lived, slow reproducing species such as the harbour porpoise, adult survival is of critical importance (Cervin et al., 2020). Based on the most recent Baltic Proper population abundance estimate of 491 individuals (Amundin et al., 2022), Carlström et al. (2023) estimated that there may be around 216 mature individuals including both females and males. Harbour porpoise populations are on average comprised of 45%–48% of females (Clausen & Andersen, 1988; Sørensen & Kinze, 1994), and a large proportion of females in the population reproduce in consecutive years (Sørensen & Kinze, 1994). However, a decline in follicles in porpoises >8 years suggested reproductive senescence, with full reproductive activity lasting ~3 years (Kesselring et al., 2018). Onset of sexual maturity in female harbour porpoises of the Baltic Sea and average age at death was calculated at 4.95 (±0.6) and 3.67 (±0.30) years, respectively (Kesselring et al., 2017), highlighting that most female porpoises in the Baltic die before they get the chance to reproduce (Kesselring et al., 2018). Despite this, performing a population viability analysis that relied on demographic and life history information from the Belt Sea population, and using a reproductive rate of 0.73, and no anthropogenic threats present, the Baltic Proper population was found to be viable, with no risk of extinction and an estimated population growth rate of 2.3% (Cervin et al., 2020). This growth rate for a harbour porpoise is alarmingly low compared to values between 4% and 9.6% reported from other harbour porpoise populations in more pristine waters in the Atlantic and Pacific (Barlow & Hannan, 1995; Forney et al., 2020; Moore & Read, 2008; NOAA, 1994; Woodley & Read, 1991). However, this value is based on a lower fertility rate of 73% from Danish waters (Sørensen & Kinze, 1994), with fertility affected by the high levels of persistent organic pollutants and detrimental effects like immune suppression and endocrine disruption on the reproductive system, which impacts population viability (Section 3.1.3) (Kesselring et al., 2018; Sonne et al., 2020, 2023). However, more population‐specific life history data from the Baltic Proper are needed to improve modelled population trajectories.

### 2.4 Species distributional range and pattern

The HELCOM indicator *Distribution of harbour porpoises* (HELCOM, 2023c) evaluates whether the distribution of harbour porpoises in the Baltic Sea is adversely affected due to anthropogenic pressures, and thus, if their distributional range and pattern are in line with prevailing physiographic, geographic and climatic conditions.

Review of old newspapers in Sweden and Finland, and summary of available information on historic records of the species in other countries, has confirmed that in the early 1900s, harbour porpoises were regularly observed in the entire Baltic Sea, including the Gulfs of Bothnia, Finland and Riga, and the Baltic Proper (Figure 1) (HELCOM, 2022). In the latter half of the 1900s, the populations range was reduced considerably, due to direct catches and bycatch (Koschinski, 2001). Currently, the species is rarely observed in the Baltic Proper, and even less so in the northern and eastern parts of the Baltic Sea (HELCOM, 2022; Koschinski, 2001).

**FIGURE 2:**
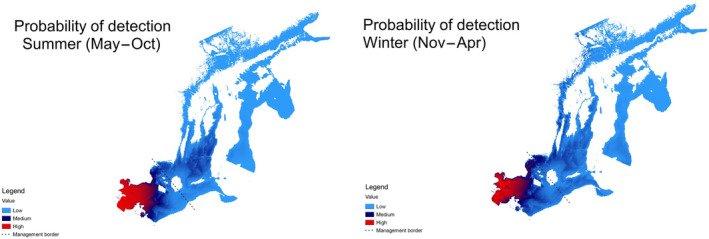
The summer (May–October, left) and winter (November–April, right) detection probabilities of the Baltic Proper harbour porpoise (*Phocoena phocoena*), as collected during the SAMBAH project (Carlén et al., 2018).

Based on the results of the SAMBAH study, the Baltic Proper harbour porpoise population is believed to have a seasonal movement pattern (Carlén et al., 2018) (Figure 2). During May–October, Baltic Proper harbour porpoises aggregate around the offshore banks south of Gotland and east of Öland (Figures 1 and 2), and most animals are believed to be east of the island Bornholm in the southern Baltic Sea. During November to April, detections were spread out along the coasts and archipelagos of the Baltic Proper (Carlén et al., 2018) (Figures 1 and 2).

National monitoring programmes and research projects have indicated seasonal movements (Gallus et al., 2012; Owen et al., 2021; Sveegaard et al., 2022; Swistun et al., 2019). Increased presence of harbour porpoises in the southern Baltic Sea during winter has been associated with cold air temperatures (Gallus et al., 2012) and severe ice conditions further north (Johansen, 1929; Lönnberg, 1940; Tägström, 1940; Wölk, 1969). It is not known how far west Baltic Proper harbour porpoises disperse in winter. Modelling of seasonal detection rates at acoustic monitoring stations in German waters of the Pomeranian Bay revealed two peaks in detection rates, with the peak in winter assumed to correspond with an influx of Baltic Proper animals, at a time when the neighbouring Belt Sea population is thought to congregate even further west (Gallus et al., 2012). This indicates a winter distribution of the Baltic Proper population extending at least as far west as to the offshore waters northeast of Rügen (Figure 1) (Gallus et al., 2012).

This delimitation of population distributions in the region was also confirmed by genetic analyses of genome‐wide single nucleotide polymorphisms (SNPs), that showed genetic distinction of individuals sampled on either side of this area (Celemín et al., 2023; Lah et al., 2016; Tiedemann, 2017). Additionally, a combination of satellite tag data from the neighbouring Belt Sea population and acoustic data have indicated a possible management border at 13.5° E (Sveegaard et al., 2015). A tentative management border from November to April has been proposed at 13° E (ICES, 2020b). Between May and October, there is a separation between the Belt Sea population and the Baltic Proper harbour porpoise populations with a tentative boundary along a line from the island of Hanö, Sweden to Jarosławiec, Poland (Figures 1 and 2). During recent decades, porpoises have primarily been sighted south of a line drawn approximately between latitude 60.5° N at the Swedish east coast and latitude 61° N at the Finnish west coast (Figure 1), and the ICES Working Group on Marine Mammal Ecology therefore suggested this as the current northern management border for the conservation of the Baltic Proper harbour porpoise population (ICES, 2020b). Whole genome resequencing techniques which can differentiate between SNPs of the three populations in Baltic waters have the potential to better assign stranded and bycaught specimens to the respective population and to unravel the population boundaries (Celemín et al., 2023).

### 2.5 Habitat for the species

The harbour porpoise is a highly mobile species which once occurred throughout the Baltic Sea with its range extending into the northernmost and easternmost parts of the Baltic Sea (Koschinski, 2001; HELCOM, 2022; Section 2.4). This area is very heterogeneous with respect to abiotic factors such as temperature, salinity, nutrient input and water depth, as well as biotic factors such as availability of prey (Ojaveer & Kalejs, 2008). Genotype–environment association analysis identified salinity as one major driver in genomic variation and candidate genes putatively underlying adaptation to the salinity gradient in the Baltic Sea have been identified (Celemín et al., 2023). Thus, it appears that low salinity does not represent a physiological barrier. Temperature is more likely an important factor in determining a porpoise habitat. Due to their relatively large body surface‐to‐area ratio, harbour porpoises need to compensate for thermal energy loss in cold water (Bjørge, 2003; Kanwisher & Sundnes, 1965; Kastelein et al., 1997; Yasui & Gaskin, 1986). Ice cover limits the occurrence in winter due to a need to access the surface to breathe (Koschinski, 2001).

It is still unclear which environmental variables drive porpoise habitat selection in the Baltic Sea. Consequently, there is currently no defined indicator for the habitat quality of Baltic Proper harbour porpoises. In order to understand the quality of available habitat for a species, it is first essential to understand which type of habitat is required by the species. Due to their high‐energy requirements, the availability of quantitatively and qualitatively sufficient prey is likely one of the most important factors for a species which must feed constantly to maintain its high canmetabolic rate. In the Belt Sea and the Kattegat, it has been shown that porpoise occurrence is correlated with their prey (Sveegaard et al., 2012). Local prey abundance can be driven by hydrographic features such as fronts, upwellings or deep channels. Such areas with a high productivity have the potential to provide important porpoise habitat. In contrast, deep basins in the Baltic Sea are hypoxic or anoxic and are less likely to represent habitat for harbour porpoises if prey are unavailable (Almroth‐Rosell et al., 2021; Krapf et al., 2022). However, the deeper waters of the Baltic Proper have not been monitored for the presence of harbour porpoises (Amundin et al., 2022).

Additionally, certain types of sediment can also be part of their habitat (Schneider von Deimling et al., 2023). Harbour porpoises are known to show bottom‐grubbing behaviour when foraging on benthic fish species (Lockyer, 2000; Schneider von Deimling et al., 2023). However, it is unclear if the Baltic Proper population use this behaviour in the acquisition of prey, due to differences in the shape of the rostrum, suggesting a more pelagic feeding strategy in the Baltic Proper compared to a more benthic in the Belt Sea (Galatius et al., 2012). In conclusion, there are likely differences in the habitat use of animals from different populations in different environments; however, there are knowledge gaps about habitat use for all Baltic Sea populations.

## 3 THREATS TO THE POPULATION AND CLASSIFICATION OF PRESSURES

**FIGURE 3:**
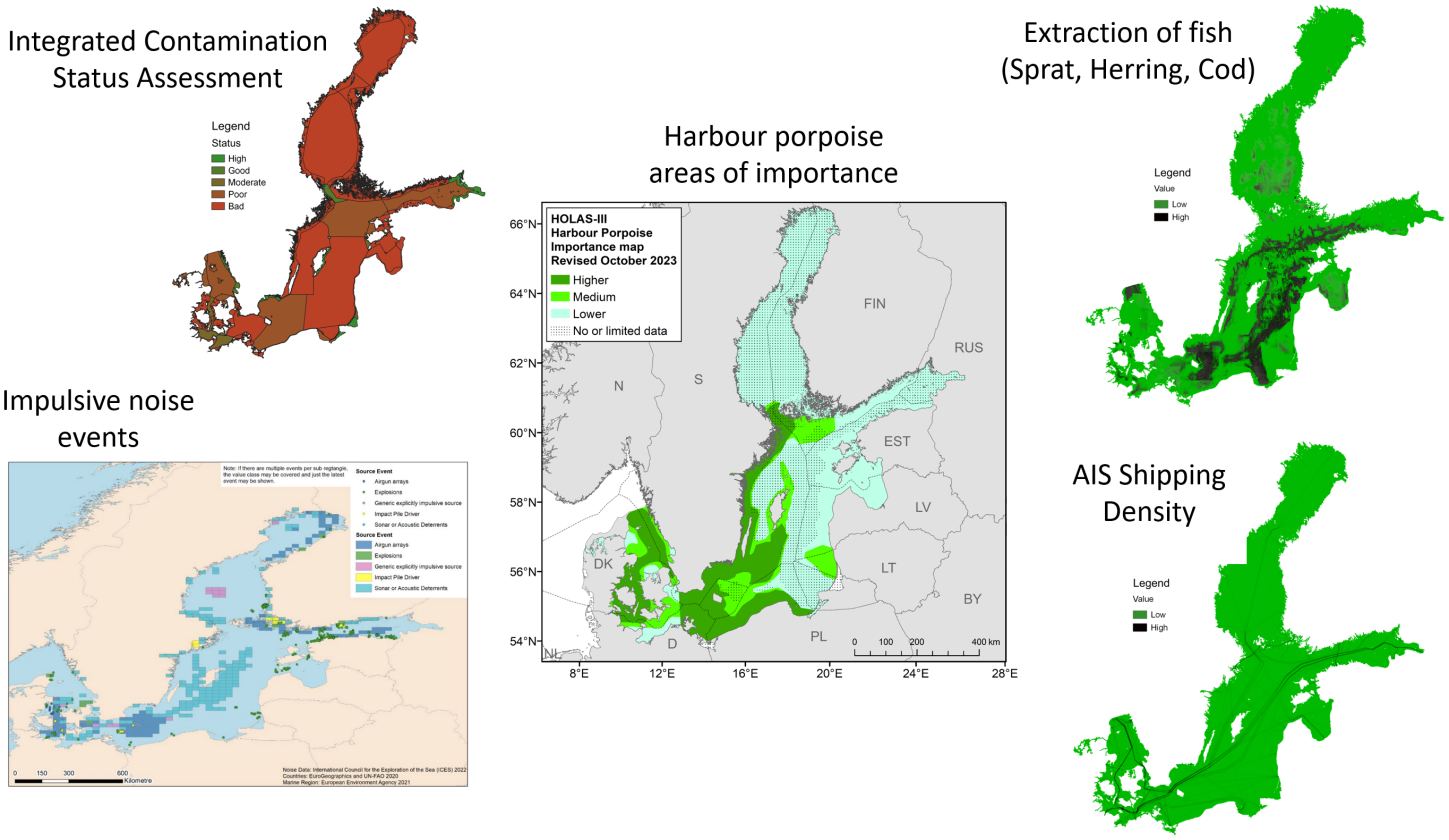
Areas of importance for the Critically Endangered Baltic Proper harbour porpoise (*Phocoena phocoena*) population (centre) (Sveegaard et al., 2022) developed as a part of the Baltic Marine Environment Protection Commission's third holistic assessment of the Baltic Sea (HOLAS 3) are shown, relative to the location of threats and pressures on the population that were identified through the same HOLAS 3 process. Major pressures (contaminants (HELCOM HOLAS 3, 2023), impulsive noise (HELCOM, 2023e)) are shown on the left and significant pressures (fish extraction as a proxy for prey removal/limitation (combined for cod (*Gadus morhua*), herring (*Clupea harengus*) and sprat (*Sprattus sprattus*) (HELCOM HOLAS 3 Dataset, 2023a, 2023b, 2023c, respectively)) and the location of shipping lanes as a proxy for continuous noise (HELCOM Dataset, 2023) are on the right. The location of fishing effort resulting in high bycatch risk is not shown due to poor quality data and lack of availability from the relevant fisheries and vessel sizes.

The harbour porpoise is a highly mobile species that uses specific areas in different seasons (Figure 2), which makes it susceptible to a large range of threats. We here define a threat to the population as an activity (e.g. gillnet fishing) and a pressure on the population as the outcome of that activity (i.e. bycatch or reduced prey availability). The Baltic Proper population is restricted to a transboundary and comparably small geographic range resulting in an increased relative exposure to human impacts and reduced natural refuge areas. Temple et al. (2024) show that extinction risk is higher for such species or populations compared to those with a wide distribution which benefits from more natural refuges. HELCOM (2023d) analysed the spatial distribution of anthropogenic pressures in the Baltic Sea and identified important areas for harbour porpoises in the Baltic Sea (Sveegaard et al., 2022) (Figure 3). Below, we summarise what is known about the impact of each pressure on the population, identify gaps in knowledge affecting its conservation, and make recommendations for future management and research (summarised in Table 1).

**TABLE 1:** Summary information on the recommendations for management action and future data collection on the population information required to assess the Baltic Proper (BP) harbour porpoise (hp) (*Phocoena phocoena*) under various European Union legislation, as well as for the major and significant pressures on the population.

Information useful for recovery management	Available for BP hp	Available other hp populations	Urgency	Recommendations for management action	Is more information needed prior to action?	Recommendations for future data collection
*Population information*						
Mortality	No	Partially available	High	Given the difficulties associated with robustly assessing the level of anthropogenic mortality, address all sources of anthropogenic mortality by implementing effective mitigation measures in the whole distribution area	No	For the Baltic Proper harbour porpoise, more information on bycatch rate of harbour porpoises through monitoring programmes would be useful. Unavoidable explosions should require a post detonation survey
Population abundance estimate	Yes	Yes	NA		No	
Recent (<6 year old) population abundance estimate	No	Yes	High	There is an urgent need for an updated abundance estimate of the population to inform on whether current management actions are sufficient and having an impact.	No	The initiation of a framework to ensure that regular (at least once every assessment cycle—6 years) abundance surveys are planned and completed is essential.
Trend in population abundance	No	Yes	Low	There is currently only one estimate of population abundance, preventing the determination of a trend in abundance over time. The initiation of a framework to ensure that regular (at least once every assessment cycle—6 years) distribution surveys are planned and completed is essential.	No	Once regular surveys for abundance have been completed, the available data will allow for a trend estimation.
Demography	No	Yes	Medium	Currently, demographic data from neighbouring populations forms the basis for population trajectories. The number of freshly dead specimens could be increased by strengthening stranding networks and educating fishermen.	No	Demographic data derived from freshly bycaught and stranded animals would improve modelled population trajectories.
Population distribution information	Yes	Yes	NA		No	
Recent (< 6 year old) population distribution information	No	Yes	High	There is an urgent need for updated information on the distribution of the population to inform on the places where protection of the population from threats would be most efficient.	No	The initiation of a framework to ensure that regular (at least once every assessment cycle—6 years) distribution surveys are planned and completed is essential.
Habitat quality	Partially available	Partially available	Medium	Improvement of the habitat, for example, by restoring prey availability or reducing disturbance would increase the potential for recovery and resilience of the population.	No	No defined indicator for harbour porpoise habitat quality is available. However, in the frame of the MSFD some attributes of their habitat are assessed. This can possibly be used for a porpoise habitat model.
*Major pressures*						
Bycatch in fisheries	No	Partially available	High	Bycatch is the main pressure and requires immediate and effective action to reduce mortality to zero. There are enough data to know where to put bycatch mitigation measures (from SAMBAH and national acoustic monitoring activities). In addition, bycatch mitigation measures that can be used are available or in a test phase.	No	For the Baltic Proper harbour porpoise, more information on bycatch rate of harbour porpoises through monitoring programmes would be useful. Additionally, identification of important bycatch risk areas through improved data on fishing effort for small vessels below 12 m and updated porpoise distribution maps would be useful. The development of effective bycatch mitigation measures that reduce the impact on fisheries would also be useful to increase the chances that sufficient protective measures for harbour porpoises are put in place.
Impulsive underwater noise	No	Partially available	High	Avoid unprotected underwater explosions. There is a need to regulate and limit the use of emerging noise sources such as seal scarers and acoustic antifouling devices. There are enough data to know that impulsive underwater noise and explosions are a high threat to the Baltic Proper harbour porpoise. Measures to protect harbour porpoise against impulsive noise are available (e.g. bubble curtains—especially for pile driving).	No	More research on effective protection measures from large scale explosions. Determination of the efficacy of standard military mitigation methods related to the impact radius as a function of charge size. Quantify the population‐level effects from frequent disturbances due to impulsive noise using, for example, agent‐based models. Due to repercussions of disturbance on the energy budget of a harbour porpoise it is also needed to collect data of prey occurrence and increase research on energetic compensation. Understand the cumulative impact of multiple impulsive noise sources. Determine the impact of wide‐spread use of echosounders. Research on novel mitigation methods for installation of infrastructure based on prolongation of the impulse (such as BLUE piling) and/or alternative low‐noise foundation methods. Research on novel geophysical survey methods to replace airguns.
Contaminants	No	Partially available	High	There is a general need to avoid contaminants and waste entering the marine environment as they negatively influence harbour porpoise health, reproduction and survival.	No	Expansion of strandings networks to ensure that rare specimens are submitted and post mortem investigations are completed. Increased understanding of the relationship between contaminant loads and their effects/risk of disease. Increased understanding of the concentrations and impact of contaminants of emerging concern
*Significant pressures*						
Prey depletion	No	Partially available	Medium	The Baltic Proper food web is distorted which can impact prey quality and quantity for the Baltic Proper harbour porpoise. It is important to introduce ecosystem‐based sustainable management of fisheries, in order to restore and maintain a functioning food web and a healthy Baltic Sea.	No	Expansion of strandings networks to ensure that rare specimens are sampled so that the diet composition of the Baltic Proper population can be determined. Collection of data on prey availability (quality and quantity) over time. Modelling energetic intake to help understand when limitations in prey availability & quality begin to impact individuals and the population.
Continuous underwater noise	Yes	Yes	Medium	The impact of disturbance including missed opportunities, displacement, acute and chronic stress on harbour porpoise health, reproduction and survival cannot be assessed at the moment. However, there is evidence that continuous noise impacts porpoise behaviour which can have repercussions for the population.	No	Data collection and modelling of continuous noise from noise sources other than commercial ships (e.g. from recreational vessels). Quantify the population‐level effects from frequent disturbances due to vessel passages using, for example, agent‐based models. Include higher frequencies more relevant for harbour porpoises in the indicator assessment and develop noise impact indicators. Due to repercussions of disturbance on the energy budget of a harbour porpoise it is also needed to collect data of prey occurrence and increase research on energetic compensation. Identify the most disturbing vessels by determining whether avoidance is impacted most by (1) specific frequencies in the spectrum, (2) the whole spectrum as perceived by the animal or (3) the perceived movement of the noise source.
Infectious disease	Partially available	Yes	Medium	Extend stranding networks and post mortem investigations to diagnose infectious pathogens and allow for swift detection of disease dynamics.	No	Systematic collection of bycaught and stranded porpoises and post mortem investigations with harmonised collection of parameters across Baltic countries.

*Note*: The urgency of action required to take management action is colour coded as high (dark grey), medium (light grey) and low priority (white). Note, action is needed in all of these areas, so white does not denote a lack of need for progress to aid in conservation and management.

For effective conservation, it is important to address threats resulting in direct mortality or reducing individual fitness which can have repercussions for vital rates and reproduction. Small cetacean populations are vulnerable to low levels of non‐natural mortalities because of their slow growth, late maturity, and low reproductive rates (Wade, 1998). As a consequence, the low number of harbour porpoise individuals in the Baltic Proper population implies that each anthropogenic mortality event impacts the population viability significantly.

In order to assist managers in prioritising actions, a classification system was used to rank pressures. Specifically, pressures were given a ranking of major, significant or minor pressure according to the following definitions:

- Major = evidence or strong likelihood of negative population effects, mediated through effects on individual mortality, health and/or reproduction;
- Significant = evidence or strong likelihood of impact at individual level on survival, health or reproduction but effect at population level is not clear;
- Minor = possible negative impact on individual fitness but evidence is weak and/or occurrences are infrequent.

Minor pressures are only briefly mentioned, as action for management should be prioritised around reducing major and significant pressures first. However, pressures ranked as minor could add to the cumulative probability of decline and need also to be taken into account and addressed.

### 3.1 Major pressures on the population

#### 3.1.1 Bycatch

##### Overview of the impact of bycatch and implications for management

Additional anthropogenic mortality has a direct impact on small cetacean populations due to their late maturity and low reproductive rates, and more so in declining or depleted populations (Kesselring et al., 2018). Thus, bycatch is ranked as a major pressure (ICES, 2019). Although no recent studies of Baltic Proper harbour porpoise bycatch are available, it is possible to draw general conclusions from historical knowledge from the Baltic Proper and at the species level as long as the same type of fishing gear is involved in available studies.

Historically, harbour porpoises were hunted, and bycaught in fishing gear, throughout the distribution range of the Baltic Proper population (HELCOM, 2022). Historic catch and bycatch data are incomplete and are considered as minimum numbers. Data from Polish fisheries inspectors' reports show that in the area around the Hel Peninsula and Puck Bay (Figure 1), at least 691 harbour porpoises were bycaught from 1922 to 1937. Most bycaught animals were recorded in drift nets, targeting salmon (638 porpoises) and the remaining ones were recorded in static nets (Psuty, 2013). On a basis of periodic reports (MFOs) sent by the inspectors to the relevant ministry, during 1934–1935, the minimum number of bycaught harbour porpoises in the same fishery was about 400, and the total number was estimated to be 800 (Psuty, 2013). From November 1960 to October 1961, at least 50 harbour porpoises were bycaught in the waters from Hanö Bight to Gotland where a driftnet fishery for salmon took place (Lindroth, 1962).

Today, similar to many other small cetacean species (Brownell et al., 2019; Temple et al., 2024), bycatch in fishing gear remains the most significant pressure to the Baltic Proper harbour porpoise (ICES, 2019, 2020a). The majority (at least 97%) of the bycatch records in the Baltic Proper have been reported to occur in static nets, such as gillnets, entangling nets or trammel nets (Berggren, 1994; Skóra & Kuklik, 2003). In addition to static nets, harbour porpoises are also bycaught in trawl fisheries (ICES, 2020a) but in much lower numbers than in static nets.

The bycatch indicator threshold agreed upon by all Contracting Parties of HELCOM for the Baltic Proper harbour porpoise population is set to zero, based on an estimated annual Potential Biological Removal (PBR) limit of only 0.7 individuals (NAMMCO & IMR, 2019). In comparison, the number of Baltic Proper harbour porpoises bycaught in 2017 was estimated to be seven individuals. As bycatch still occurs in the Baltic Proper, this zero bycatch threshold is exceeded. GES was not achieved in the most recent HOLAS 3 assessment (HELCOM, 2023a). PBR is estimated via a simulation method to predict long‐term population development based on a simple population model under scenarios of additional potential mortality (Wade, 1998). The current bycatch level is a serious threat to the population, especially due to the low numbers of individuals participating in reproduction (Kesselring et al., 2018).

##### Gaps in knowledge on bycatch affecting conservation

The major data gaps on bycatch of harbour porpoises are divided into three elements:

1. Lack of bycatch monitoring (through monitoring of fishing activities or cause of death of stranded animals) and reporting by fishermen and other stakeholders, preventing accurate estimates of bycatch rate.
2. Imprecise and incomplete reporting of data on fishing effort (especially at a relevant spatiotemporal scale and for small vessels below 12 m), which prevents calculations of total bycatch and identification of areas of most intense fishing activity.
3. Lack of updated information on the distribution of harbour porpoises, preventing identification of the areas of highest overlap with fisheries where there is the largest risk of bycatch for the population.

These knowledge gaps are also highlighted in the *HELCOM Roadmap on fisheries data* (HELCOM, 2020).

ICES collects information on bycatch of protected species from various monitoring programmes under EU legislation (mainly EU Technical Regulation 2019/1241) and scientific monitoring programmes (currently mainly under the EU Data Collection Framework, DCF). ICES (2017) states that bycatch observations ‘are insufficient to enable any assessment of the overall impact of EU fisheries on [marine mammals]’. This has been reiterated (e.g. ICES, 2022a) but not acted upon. Sampling under the current DCF can contribute to the assessment of bycatch of Protected, Endangered and Threatened Species (PETS), but is insufficient on its own as currently implemented by Member States. DCF sampling focuses on discards and mainly on fishing gears (e.g. towed gears) which are not a major concern for porpoise bycatch. Since the current national DCF monitoring programmes only target marine mammal bycatch to a limited extent, on‐board monitoring of fishing operations with static nets (with high bycatch risk to harbour porpoises) is still low (ICES, 2022b). In addition, EU Regulation 2019/1241 obliges countries to monitor bycatch of cetaceans only on fishing vessels with an overall length of 15 m or more, and thus has the focus on the wrong fleet segment to monitor porpoise bycatch. Assessments carried out by the ICES Working Group on Bycatch of Protected Species (ICES, 2018) demonstrated that bottom trawling is generally oversampled with respect to monitoring of protected species bycatch, and passive gear types (e.g. fyke nets, trammel nets, set gillnets, set longlines, pots and traps) are under‐sampled in the Baltic Sea (ICES, 2018, 2019). Among these under‐sampled gears are those which represent the highest bycatch risk for the Baltic Proper harbour porpoise population. Therefore, the lack of appropriate bycatch monitoring programmes despite legislation requiring monitoring of bycatch in all relevant fisheries prevents an assessment of the bycatch rate.

Further, data on fishing effort are necessary to estimate the total bycatch. To calculate the total bycatch, at a minimum, an estimate of the bycatch rate expressed as the number of animals bycaught per unit of fishing effort (BPUE) for each gear is multiplied by the total fishing effort of the relevant fleet in the area. The EU Control Regulation specifies what type of fishing vessel‐tracking system is mandatory for various fleet segments and how fishing effort shall be reported. Until the end of 2023, only vessels ≥12 m in length had to be equipped with a Vessel Monitoring System (VMS) and an electronic logbook. Vessels >10 m in length (> 8 m in the Baltic Sea when they had a cod quota (According to Reg. 2016/1139)) had to have a logbook. Smaller vessels were not required to carry a logbook or fill out a landing declaration. For smaller vessels, fishing effort was derived by individual Member States as monthly journals (Germany), coastal logbooks (Sweden), sales records (Denmark) or extrapolated sampling data, which do not provide data on fishing effort that are precise and comparable for estimation of total bycatch of harbour porpoise. Fishing effort was collected as ‘days at sea’ which is not precise enough to ensure robustness of extrapolations (from bycatch rate per effort to total bycatch) for static nets (Moore et al., 2021). The preferred metric of fishing effort would be ‘total soak time of nets in kilometre hours’ for the observed effort.

In order to address the lack of necessary data and poor data quality, the EU Control Regulation (2023/2842) was adopted in January 2024. From January 2028, all vessels shall have a fully functioning tracking device. As an alternative to a system permanently installed on a vessel, vessels <12 m may now carry a mobile tracking system, such as a tracking mobile phone app. However, derogations until 31 December 2029 are possible for some vessels <9 m which are not subject to restrictions, applicable in any fishing restricted area in which they operate. In much of the distribution range of the Baltic Proper harbour porpoises, there are areas in which no fishing restrictions apply, meaning valuable data on fishing effort may still be missing until 2030. Additionally, the inaccuracy of the effort metric ‘days at sea’ has not been resolved in the new Regulation. As small vessels are the fleet segment that dominates fishing with static nets, they should be the main focus of fishing effort data collection, and any harbour porpoise bycatch monitoring programme. However, currently the effort data available from this segment still has the lowest quality of all.

The lack of information on fishing effort also prevents locating high‐risk bycatch areas for the Baltic Proper harbour porpoise with sufficient confidence. Areas of high bycatch risk, which should be the focus areas for mitigation measures, are largely unknown. The HELCOM ACTION project (HELCOM, 2021b) provided initial data on high bycatch risk areas in parts of the Baltic Sea on the basis of spatiotemporal data on relative detection rate of harbour porpoise (taken from the SAMBAH data from 2011 to 2013, http://sambah.org/) and available data on relevant fishing effort. It identified areas where monitoring of bycatch needs to be intensified, or where preventive measures could be focused in order to have the best effect. However, there are limitations in the results largely due to the limited availability of fishing effort data described above. It is desirable to update bycatch risk maps using improved fishing effort data that may result from the new EU Control Regulation. These data, combined with updated data on the distribution of the Baltic Proper harbour porpoise from the SAMBAH II project could be used to further update information for managers, once available. Adaptive management that acts now to implement conservation measures, and then updates these measures when new information is available is essential to protect species from major pressures such as bycatch.

#### 3.1.2 Impulsive noise

##### Overview of the impact of impulsive noise and implications for management

Impulsive noise can be defined as a sound emitted by a point source comprising one or more sounds of short duration and with longer gaps between these pulses. The EU MSFD Descriptor 11 (Energy and Noise) is divided into two criteria; low‐ and mid‐frequency impulsive sound sources (D11C1) and continuous low‐frequency sound (D11C2) (see Section 3.2.2). Criterion D11C1 states as follows: ‘The spatial distribution, temporal extent, and levels of anthropogenic impulsive sound sources do not exceed levels that adversely affect populations of marine animals’. Its interpretation of low‐ and mid‐frequency impulsive sound, is sound of an effective time duration of individual pulses of less than 10 s, and whose repetition time exceeds four times the effective duration (i.e. with a duty cycle of 25% or less), or single sound events of less than 10 s duration in a frequency range of 10 Hz to 10 kHz (Van der Graaf et al., 2012). It is important to emphasise that regarding this criterion, only population‐level effects are to be avoided. In order to predict auditory effects from impulsive sound on marine animals, dual exposure criteria have been proposed as frequency‐weighted sound exposure level (SEL) and peak sound pressure (Southall et al., 2019). However, impulsive sound may also differ with respect to signal duration and rise time, intervals, and ‘impulsiveness’ (Guan et al., 2022; Southall et al., 2007; von Benda‐Beckmann et al., 2022).

Harbour porpoises produce narrow‐band high‐frequency clicks with a peak frequency around 130 kHz (Au et al., 1999; Macaulay et al., 2020; Villadsgaard et al., 2007). Listening to the echoes of these clicks can provide animals with information on the location, size, and acoustic density of nearby prey, but also allows them to spatially orientate (Verfuß et al., 2005). In foraging ‘buzzes’, the inter‐click interval is reduced as the porpoise approaches prey (DeRuiter et al., 2009). Similar click trains are used in social interactions, particularly between mothers and calves (Koschinski et al., 2008; Sørensen et al., 2018). Therefore, harbour porpoises rely on sound production and hearing to communicate with conspecifics, avoid threats, navigate, and to find prey.

There are no studies related to noise focusing on the Baltic Proper harbour porpoise population.  It is feasible to draw conclusions on species hearing based on knowledge from other populations, as hearing likely evolved over longer time‐scales than the existence of the Baltic Sea. However, conclusions relating to sound propagation require some caution due to reverberation effects in the shallow or stratified waters of the Baltic Sea (Pihl et al., 2011; Sigray et al., 2016) and differences in salinity affecting absorption (Richardson et al., 1995), which could produce some bias in effect distances. Effects of impulsive noise on harbour porpoises can range from acoustic disturbance, to temporary (TTS) or permanent hearing threshold shifts (PTS), or even physical injuries and mortality (Lucke et al., 2009; Schaffeld et al., 2020; Siebert et al., 2022). Harbour porpoises' acoustic sense has evolved to be their likely dominant sense vital to their survival. Any impairment or damage to their auditory system has deleterious consequences for the affected individuals (Lucke et al., 2009). In a small, critically endangered population, direct impacts on the viability or survival of even a few individuals will have negative repercussions for the entire population. Further, a number of sound sources are known to displace animals over large distances (Brandt et al., 2011; Dähne et al., 2013, 2017; Richardson et al., 1995; Thompson et al., 2013; Tougaard et al., 2009) or to interfere with their feeding behaviour at large ranges which may have energetic consequences (Pirotta et al., 2014; Sarnocińska et al., 2020; Todd et al., 2022). Therefore, impulsive noise is rated a major pressure on the Baltic Proper population (ICES, 2019).

Anthropogenic activities producing impulsive sound can create sound either intentionally as a part of the activity (such as seismic surveys or sonar), or as a by‐product of the activity (such as explosions or pile driving). HELCOM requires Contracting Parties to report impulsive noise sources to the impulsive noise registry (https://www.ices.dk/data/data‐portals/Pages/impulsive‐noise.aspx), a database held by ICES for the purpose of MSFD assessment.

With respect to an animal's vulnerability to noise, the spectral characteristics of the noise are highly important. For both injury and disturbance, frequency weighting with respect to porpoises' hearing abilities is a way to assess noise impact and to regulate noise (Finneran, 2015; Southall et al., 2019; Tougaard et al., 2022; Tougaard & Dähne, 2017). The most recent guidance for marine mammals in order to avoid temporary hearing loss and injury to the auditory system is given by Southall et al. (2019). Thresholds were based on levels required to induce a 6 dB TTS in experiments with captive marine mammals. Differing hearing abilities were accounted for by using specific frequency weighting functions for different functional hearing groups. These resemble inverted audiograms. For impulsive noise from pile driving or airguns there is strong support for thresholds using the weighting function for very high‐frequency hearing specialists (Tougaard et al., 2022). However, at high frequencies there are some discrepancies between measured TTS onset and the weighting function which might be explained by an ability to raise the threshold when animals anticipate high‐intensity sound at high frequencies (Tougaard et al., 2022), a mechanism known from bottlenose dolphins (Finneran, 2018).

The severity of the impact of noise on individual animals also depends on how frequently they are exposed to noise, as well as the intensity and duration of the sound. Among the loudest point source emitters are underwater explosions, for example, from clearance of unexploded ordnance (UXO) or construction work (Bellmann et al., 2020; Richardson et al., 1995). Although the noise event is very short in an explosion, due to their high level and very short signal rise time, the effects can be detrimental (Koschinski, 2011). Explosions of UXO have been linked to blast injuries and the death of harbour porpoises (Siebert et al., 2022; von Benda‐Beckmann et al., 2015). In one analysed case, blast‐induced hearing loss subsequently led to bycatch (Siebert et al., 2022). In the North Sea, a population impact of regular UXO clearing on the much larger population has been shown (von Benda‐Beckmann et al., 2015). In the Baltic Sea, approximately 175,000 mines are estimated to have been laid during the world wars of which a large fraction is expected to still be on the sea bottom, potential targets for clearing operations that often detonate the mines (Koschinski, 2011; Wichert, 2011). It can be predicted that underwater blasts will increase as legacy munitions are frequently found during surveys preparing for infrastructure and renewable energy development which is a growing industry in the Baltic Sea (See map of planned offshore wind farms at: https://map.4coffshore.com/). However, the potentially lethal result of this activity is not covered by the existing MSFD impulsive noise indicator, which only focuses on the fraction of habitat of noise‐sensitive species in which acoustic disturbance occurs.

In addition to explosions, there are many other sources of impulsive noise in the Baltic Sea. In the 6‐year period 2016–2021 (2190 days), the most commonly occurring events (days with at least one impulsive noise activity) of impulsive noise sources in the Baltic Sea were from ‘sonars or ADDs’ (1400 days—the impulsive noise registry does not distinguish between these), explosions (>800 days; only a very minor fraction was mitigated, for example, with bubble curtains) (HELCOM, 2023e), seismic surveys (600 days), and pile driving (500 days) were reported. These sources can have the following impact on harbour porpoises:

- *Sonar*. Military sonars use a wide range of pulsed sounds or sweeps of varying frequencies, durations and inter‐pulse intervals (Van der Graaf et al., 2012) making it difficult to predict the impact on harbour porpoises. Since low‐ and mid‐frequency military sonars are very powerful, significant TTS could occur during longer exposures (e.g. 30–60 min at a cumulative SEL between 175 and 180 dB re 1 μPa^2^s). This depends on the received level, exposure duration and inter‐pulse interval, with longer intervals allowing for better recovery of the ear (Kastelein et al., 2014, 2017a; Kastelein, Gransier, et al., 2015). Further, sweeps of low‐ and mid‐frequency sonar induce avoidance behaviour in harbour porpoises. This depends on frequency, received level, background noise, and sweep characteristic, as well as the occurrence of side bands (Kastelein et al., 2011; Kastelein, van den Belt, Gransier, et al., 2015).
- *Acoustic deterrent devices* (ADDs). These include pingers (used on gillnets to mitigate bycatch) or seal scarers (frequently used to scare porpoises intentionally to protect porpoises from intense impulsive noise such as piling or explosions which have a strong potential for injury. These are also increasingly deployed in fisheries to protect the catch against seal depredation (Svels et al., 2023)). These devices have some characteristics of both continuous and impulsive sound; however, due to their high source level, the impact ranges of these devices can be high (Findlay et al., 2021, 2024). Porpoises can experience TTS or PTS, show evasive responses, and are displaced over large distances with effects described up to 7.5 km away (Brandt et al., 2013; Graham et al., 2023; Olesiuk et al., 2002). The widespread use of ADDs (pingers or seal scarers) has the potential for habitat degradation or even habitat loss (Findlay et al., 2021, 2024). The use of seal scarers as a protective mitigation measure can induce hearing impairment in harbour porpoises (Schaffeld et al., 2019).
- *Seismic surveys*. Depending on the level and exposure duration, these have the potential to cause TTS or PTS and reduce hearing sensitivity in harbour porpoises (Lucke et al., 2009). During a seismic survey, no long‐term and large‐scale displacement was found, however, reduced echolocation behaviour was observed at distances of up to 12 km (Sarnocińska et al., 2020). Additionally, missed feeding opportunities have been demonstrated in harbour porpoises' response to seismic surveys (Pirotta et al., 2014). If such disturbance occurs often, in larger areas or for long periods, this can have energetic consequences for the individual given the fact that porpoises have high energetic requirements (Rojano‐Doñate et al., 2018, 2024). This can affect the nutritional state, survival or reproductive success and thus have negative population consequences (National Research Council, 2005).
- *Pile driving* (i.e. mostly in association with the construction of offshore wind farms). Considerable displacement has been shown during the construction of offshore wind farms in the North Sea, due to piling noise in combination with the use of seal scarers (Brandt et al., 2016; Dähne et al., 2017; Schaffeld et al., 2019; Tougaard et al., 2009). The zone of displacement during impact piling has been shown to extend over 20 km (Tougaard et al., 2009). Depending on the received level, such displacement can last for up to 2 days until animals begin returning to an area (Brandt et al., 2016). Multiple exposures to pile driving noise were shown to potentially have effects on harbour porpoise hearing (Schaffeld et al., 2020).
- *Hydroacoustic research equipment*. Such as sediment profilers, multibeam or sidescan sonars, airgun arrays, boomers or sparker, etc., also typically applied in the pre‐construction phase of offshore renewable development, has a wide range of signals with respect to source level, directionality, frequency content, sweep characteristic, duration and duty cycle (Ruppel et al., 2022). According to the definition above, not all of them are considered in the impulsive noise registry and MSFD assessments. Although their impact on harbour porpoises has not been investigated specifically for each application, some general conclusions can be drawn. A frequency spectrum in the range of the best hearing of porpoises, omnidirectional sources, directional sources with side lobes, or high‐intensity sources have a higher potential to affect hearing or behaviour of harbour porpoises. Higher frequency signals are absorbed by sea water to a greater extent, but are in the range of better hearing of porpoises. Longer signals and larger duty cycles have a higher potential for TTS than shorter signals or lower duty cycles. High‐intensity sources have the potential to affect the hearing, cause injury and affect the behaviour whereas the impact of lower‐intensity sources may be on the behaviour only (Kastelein et al., 2014, 2017a, 2017b; Kastelein, Gransier, et al., 2015; Kastelein, van den Belt, Gransier, et al., 2015; Kastelein, van den Belt, Helder‐Hoek, et al., 2015; Richardson et al., 1995; Ruppel et al., 2022).

The distribution of low‐ and mid‐frequency impulsive sounds covered by the impulsive noise registry in the period 2016–2021 is shown in Figure 3 (HELCOM, 2021b). It is evident that much of the activities overlap with areas of high and medium importance for the Baltic Proper harbour porpoise population. Sonar and ADDs events occurred South of Gotland and East of Öland as well as in the Southwest of the Bothnian Sea. Further, there was airgun activity around Bornholm/South of Hanö Bight and a number of explosions along the Polish coast. Based on offshore wind farms under construction or in the planning stage (https://map.4coffshore.com/), potential future conflicts can be predicted in large areas of high and medium importance for harbour porpoises (Figure 3) such as South of the Åland Islands, south of the Swedish Archipelago Sea, south of Gotland, around the Midsea Banks in Swedish and Polish waters, and in the central Pomeranian Bay.

##### Gaps in knowledge on impulsive noise affecting conservation

From a regulatory perspective, there is a good understanding of auditory effects of impulsive noise in harbour porpoises from experimental studies with various delphinid species (Finneran, 2015; Houser et al., 2017; Southall et al., 2019; Tougaard et al., 2022). However, there are uncertainties in which frequency weighting represents the biological mechanisms the best and how experimental methods affect the TTS onset values (Tougaard et al., 2022). Also, it is not fully understood how multiple pulses such as in piling or seismic noise accumulates in the ear and how silence intervals between pulses need to be taken into account (Kastelein et al., 2014, 2017a; Kastelein, Gransier, et al., 2015; von Benda‐Beckmann et al., 2022). It is currently unknown how many porpoises are injured or killed by underwater explosions from UXO clearing, which has direct implications for the population. The role that hearing loss plays in entanglement is also unknown. These uncertainties call for some level of caution in management of impulsive noise in order to enable the full recovery of the Baltic Proper population.

Further, sound sources using higher frequencies than 10 kHz such as most echo sounders and fish finders are not covered by the MSFD indicator and hence are not reported to the impulsive noise registry. Thus, there is a gap in the assessment. However, it is known that these sounds can also induce aversive behaviour depending on the sound pressure level, inter‐pulse interval and occurrence of side bands (Kastelein, van den Belt, Gransier, et al., 2015; Kastelein, van den Belt, Helder‐Hoek, et al., 2015). Absorption at higher frequencies reduce effect ranges compared to lower frequencies, but in contrary, at higher frequencies, harbour porpoises have a better hearing (Kastelein et al., 2017b; Richardson et al., 1995). At similar broadband sound pressure levels, signals with side bands (multiples of the fundamental frequency) have a greater effect on the behaviour than signals without. This is attributed to the audibility of the higher frequency component in side bands but also to the way porpoises perceive a sound (Kastelein, van den Belt, Gransier, et al., 2015). Since sources using frequencies >10 kHz are omnipresent and overlap with frequencies of good hearing in harbour porpoises they also deserve attention in assessing noise effects. Accordingly, the HELCOM Regional Action Plan for Underwater Noise calls for identification of other noise sources with significant impact on marine ecosystems, assessment of their impact and actions to reduce this (HELCOM, 2021b).

Acoustic antifouling devices also fall under this category. They are an emerging threat and are increasingly used for cleaning propellers, engine‐cooling systems and even the hull of vessels by inducing vibration or cavitation on a metal surface and thus preventing the settlement of biofouling organisms. Manufacturers of such devices claim to be environmentally friendly because toxic paints can be avoided but completely disregard their noise emissions. These systems are currently unregulated and can be found in oceangoing vessels as well as in recreational vessels. Trickey et al. (2022) found that acoustic antifouling devices on oceangoing cruise liners (typically used at the sea chest or the piping system) caused clear avoidance by Cuvier's beaked whales (*Ziphius cavirostris*). They detected a fundamental frequency of around 20 kHz with harmonics and also pulses up to a frequency of 166 kHz, although sound pressure level measurements are not available (Trickey et al., 2022). These frequencies cover porpoises' best hearing (16–140 kHz), and thus widespread use of these devices has the potential for deterring harbour porpoises from vessels equipped with such devices (and possibly also causing TTS). If this happens too often, this affects energetics and has the potential for population consequences (Rojano‐Doñate et al., 2018, 2024). A first step in filling the gap would be noise measurements of devices available on the market.

The impact of impulsive noise on individual harbour porpoise hearing and behaviour have been intensively studied (e.g. Kastelein, van den Belt, Gransier, et al., 2015; Kastelein, van den Belt, Helder‐Hoek, et al., 2015; Lucke et al., 2009; Schaffeld et al., 2020; Siebert et al., 2022; Southall et al., 2019); however, the repercussions at the population level are uncertain; an essential gap to fill as the MSFD criterion on impulsive noise only addresses population‐level effects. At present, it is difficult to accurately quantify population‐level effects from frequent disturbances, which can be predicted by means of agent‐based models still under development (Nabe‐Nielsen et al., 2018). Such models require a high level of detailed knowledge on porpoise energetics and the energetic consequences of disturbance, which has been formulated as a research priority (Sarnocińska et al., 2020).

Impulsive noise is assessed by the HELCOM indicator on the ‘Distribution in time and place of loud low‐ and mid‐frequency anthropogenic impulsive sounds’ (HELCOM, 2023e). Although some initial thresholds were set for HOLAS 3, it is not clear what thresholds are required to avoid population‐level consequences for the Baltic Proper harbour porpoise. This indicator only addresses the pressure but not the impact of underwater noise on noise sensitive species such as the harbour porpoise. Such an impact indicator would also need to take into account the animals injured or killed by impulsive noise, which is currently not covered by the impulsive noise indicator. The need to develop noise impact indicators has been highlighted in the Baltic Sea Action Plan (Action S62, HELCOM, 2021a) and HELCOM Recommendation on the Regional Action Plan on Underwater Noise (Action 5, HELCOM, 2021b).

According to MSFD D11C1, Member States shall establish threshold values for spatial distribution, temporal extent and levels of anthropogenic impulsive sound sources, taking into account regional or sub‐regional specificities. One of these specificities is the occurrence of the Critically Endangered Baltic Proper harbour porpoise population, which, due to its status, requires a high level of precaution, especially in the light of knowledge gaps and the absence of impact indicators as outlined here.

Offshore development is still limited but increasing in the Baltic Sea and there are extensive plans for the development of offshore wind farms (https://map.4coffshore.com/). Activities linked to different phases of the life cycle of an offshore wind farm generate impulsive noise, for example, seabed exploration, explosions for clearance of cable corridors and wind farm areas, pile driving and possibly decommissioning. Another sector of future offshore industrialisation is carbon capture and storage, which will require seismic for exploration, monitoring and maintenance. Environmental impact assessments only assess the impact of a single project, but the cumulative impact of all projects remains unclear.

Echo sounders and fish finders produce lower levels of impulsive noise compared to piling or airguns, but are ubiquitous in the Baltic Sea. Further, some of them are operating at the frequencies of the best hearing for harbour porpoises (Ruppel et al., 2022). Even those operating at 200 kHz (above the audiogram for a harbour porpoise) radiate sub‐harmonic frequencies around 100 kHz in their side lobes; these are audible to porpoises despite their inaudible fundamental frequency (Deng et al., 2014). Although the range of high‐frequency echo sounders may only be a few hundred metres and individual effects of single exposures can be neglected, the overall extent of the disturbance caused by these widely used devices should be investigated.

With increasing industrial and military activities, mitigation of noise becomes critical for the conservation of noise‐sensitive species such as the harbour porpoise. Some effective mitigation measures, such as a combination of seal scarers and noise abatement (e.g. bubble curtains which can reduce the impact of impulsive noise on hearing (Dähne et al., 2017)) are available. OSPAR Commission (2016) presented a review of effective mitigation methods for piling. However, increasing turbine size requires larger monopiles, which radiate higher noise levels during piling (von Pein et al., 2022). This means that it may be impossible to meet the current standards in the near future (Bellmann et al., 2020). Research on novel mitigation methods based on prolongation of the impulse (making it less 'impulsive') and especially alternative low‐noise foundation methods needs to be intensified. Military mitigation standards are not up‐to‐date as Siebert et al. (2022) were able to show. Currently, in UXO clearing, Baltic Sea Navies rely on observers and deterrent methods such as small pre‐charges and one seal scarer (Hermannsen & Tougaard, 2014). Given the high‐pressure levels and large possible impact ranges of UXO detonations of up to many kilometres (Robinson et al., 2022; von Benda‐Beckmann et al., 2015), it is surprising that no systematic study on the efficacy of standard mitigation methods as a function of, for example, charge size is available. The efficiency of pre‐charges has never been investigated but these may pose a risk themselves (Koschinski, 2011).

So far, no systematic noise measurements from various types (continuous, pulsed, frequency, harmonics) of acoustic antifouling devices are available and it remains unclear how porpoises react to these and what mechanisms are relevant. Individual energetic and population consequences of disturbance by these devices are unknown. Further, the potential for TTS (or even PTS in repeated exposure during potential widespread use) caused by transiting ships equipped with these devices is unclear and is especially important for SAC management where shipping lanes traverse the SACs (see Figure 1 and Figure 3).

#### 3.1.3 Environmental contaminants

##### Overview of the impact of environmental contaminants and implications for management

Pollutants are classified as a major pressure to harbour porpoises (ICES, 2019) because of their impact on health, fertility and reproduction. They are considered a factor in the decline of the Baltic Proper harbour porpoise (HELCOM, 2013; Kannan et al., 1993; Koschinski, 2001). Exposure to contaminants, and thus contaminant burden and composition in porpoise tissues, differ between regions (Huber et al., 2012). Unfortunately, limited contaminant data for the Baltic Proper harbour porpoise population are available due to a shortage of samples. However, some studies focusing on other organisms in the geographic area where this population occurs are available and these are valuable to predict effects on the population (de Wit et al., 2020; Kannan et al., 1992; Karl & Ruoff, 2007; Potrykus et al., 2003). From other areas, general principles such as bioaccumulation and toxicity in harbour porpoises can be deducted (Weijs et al., 2009, 2010).

Cetaceans are high trophic level foragers and exposed to a high bioaccumulation of contaminants. Biomagnifying pollutants such as persistent organic pollutants (POPs; e.g. polychlorinated biphenyls (PCBs), dioxins, perfluoroalkylated substances (PFAS)) and heavy metals (e.g. mercury) are of particular concern (Weijs et al., 2007). They may act as endocrine disruptive chemicals, affecting the reproductive system, thyroid gland, neuroendocrine system, immune system, and the systems that control nutrient partitioning (Beineke et al., 2005; Hall et al., 2006; Lehnert et al., 2019; Murphy et al., 2015; Williams et al., 2020).

Hazardous substances are entering the Baltic Sea via different routes. Direct discharges, inputs via rivers, atmospheric inputs, as well as sea‐based sources are of importance. Inputs from Wastewater Treatment Plants (WWTP), runoff from agriculture or urban areas, industrial emissions and discharges from shipping lead to the Baltic Sea being polluted by a wide variety of hazardous substances (HELCOM, 2023f). An emerging source of contaminants is the increasing use of exhaust gas cleaning systems (scrubbers) in commercial vessels which effectively washes heavy metals and Polycyclic aromatic hydrocarbons (PAH) into the marine environment, especially if operated open‐loop (Claremar et al., 2017; Endres et al., 2018). In 2024, Denmark became the first country in the Baltic Sea to ban open‐loop scrubber discharge in their coastal waters from 1 July 2025 and closed‐loop scrubber discharge from 1 July 2029 onwards (https://safety4sea.com/denmark‐prohibits‐scrubber‐discharge‐in‐its‐waters/) which may mark a turning point in the Baltic Sea. Different types of PFAS are used in many products, from makeup and clothing, to cleaning detergents and fire‐fighting foams (Fair & Houde, 2018). Further, legacy contaminants such as DDT and PCBs remain in the ecosystem for a long time after being banned or restricted (Stuart‐Smith & Jepson, 2017).

In harbour porpoises, high burdens of PCBs were found to be associated with reduced immune system function, health status and fertility (Beineke, Siebert, Müller, et al., 2007; Beineke, Siebert, Stott, et al., 2007; Jepson et al., 2005; Murphy et al., 2015). Relatively low PCB toxicity thresholds for onset of physiological impacts (9 mg/kg lipid as ∑PCB) in marine mammals and the highest PCB toxicity threshold reported for profound reproductive impairment in ringed seals (*Phoca hispida*) in the Baltic Sea (41 mg/kg lipid as ∑PCB) were used for comparing PCB concentrations in harbour porpoises and other odontocetes (Jepson et al., 2005). Organotin compounds (OTCs), commonly used as antifouling in marine paints because of their biocide properties were analysed in Polish harbour porpoises revealing a significant degree of tributyltin pollution and higher concentrations than in porpoises from other geographic areas (Ciesielski et al., 2004). Concentrations of ∑OTCs in harbour porpoises from the North‐ and Belt Sea exceeded cytotoxic levels in one animal (n=23) and ∑7PCBs were above thresholds for adverse health effects in seven porpoises (n=22) (Roos, 2021).

Most of the data on PCB concentrations of the Baltic Proper harbour porpoise population are from the 1980s or 1990s (Berggren et al., 1999; Bruhn et al., 1999; Falandysz et al., 2002; Kannan et al., 1993) and were often well above a proposed threshold for adverse health effects (Jepson et al., 2005). Moreover, the levels were up to 254% higher than mean levels of PCBs in corresponding samples from Kattegat and Skagerrak (Berggren et al., 1999). Toxic equivalence (TEQ) values of dioxins, dioxin‐like PCBs and chloro‐organic contaminants in herring (*Clupea harengus*) fillets sampled at 11 locations ranging from west of the British Isles to the Latvian coast in the Baltic Proper during 1996–2004 show an increase of about 35 fold from west to east (Karl & Ruoff, 2007). In the Swedish Baltic Sea, porpoises were found to have three times the level of PCBs and more than 10 times the level of DDT compared to porpoises from the Kattegat/Skagerrak Seas or Norway (Berggren et al., 1999). Moreover, porpoises from the Polish coast had high concentrations of the pesticides aldrin, dieldrin and chlordane, and their blubber also contained mirex, heptachlor and heptachlor epoxide (Kannan et al., 1993; Strandberg et al., 1998). PCB concentrations were significantly higher than thresholds for adverse health effects in 11 porpoises from Swedish waters. However, all 9 reproducing females were below the 9 ppm lw (1–7 ppm lw) adverse health effects threshhold, reflecting the potential transfer of pollutant loads via gestation and lactation to calves (Roos et al., 2024).

When considering PFAS, Baltic harbour porpoises (*n* = 37) from 1991 to 2008 had a higher contaminant burden in the liver tissue than animals from the North Sea, Iceland and the Norwegian Atlantic Ocean (Huber et al., 2012). Among the reported effects of PFAS are reproductive toxicity, neurotoxicity, immunotoxicity and hepatotoxicity (Fair & Houde, 2018) and given the persistence and bioaccumulation potential of PFAS, their toxicity to wildlife at high trophic levels is of concern (Galatius et al., 2013). A recent review on health effects of contaminants in wildlife of the Baltic Sea is available in Sonne et al. (2020).

Recently, a decrease in the concentrations of some regulated chemicals in blubber, such as in PCBs has been observed (Rebryk et al., 2022; Roos, 2015). This can be attributed to the cessation of use of these chemicals. Similarly, other compounds (e.g. perfluorooctane sulfonamde, PFOSA) were also shown to be decreasing, potentially reflecting the phase‐out of perfluorooctane sulfonate (PFOS)‐based products (Huber et al., 2012). Since the ban of PCBs in the 1980s, PCB concentrations in marine mammals initially declined worldwide, but have since stabilised at toxicologically significant levels in several European cetacean species (Law, 2014: Jepson et al., 2016). A similar temporal pattern was observed in POP compounds and their TEQ values in Baltic herring and seabird eggs (de Wit et al., 2020; European Food Safety Authority, 2005; Jörundsdóttir et al., 2006; Miller et al., 2014). A recent study in the United Kingdom (Williams et al., 2023) showed that mean PCB blubber concentrations were observed to decline in all harbour porpoise Assessment Units and OSPAR Assessment Areas in UK waters. However, a high proportion of animals were exposed to concentrations deemed to be a toxicological threat, though the relative proportion declined in most assessment units over the last 10 years. The study suggests that although PCBs were banned now more than 40 years ago, they remain in ecosystems and their bioaccumulation in marine mammals depends on trophic ecology and history of pollution at a very local scale. This stresses the fact that assessing PCB levels and its effects in marine mammals today is still relevant.

However, while legacy POPs have declined in the Baltic Sea due to bans and regulation, concentrations of new chemicals increase when entering the environment as replacements. A recent wide‐scope target and suspect screening study under the German HELCOM Chairmanship 2020‐2022 of pooled liver and muscle samples from marine mammal species, including the harbour porpoise, in the Baltic Sea identified known toxic chemicals like PCBs and DDT, as well as many sources of contaminants of emerging concern (CECs) including agricultural and industrial chemicals, pharmaceuticals, flame retardants and UV filter octinoxate (Slobodnik et al. 2022). Concentrations of 33 of these compounds exceeded their Predicted No‐effect Concentration (PNEC) values for marine fish, suggesting potential adverse health effects when bioaccumulated along the foodweb in top predators. The majority of the detected chemicals were registered under the EU Regulation on the registration, evaluation, authorisation and restriction of chemicals (REACH) indicating their annual high tonnage production (Slobodnik et al., 2022). Seven out of eight CEC detected in harbour porpoise tissue showed increasing trends of up to 19% between 1998 and 2019 (Rebryk et al., 2022). Liver, blubber and muscle tissue of harbour porpoises (*n* = 6) from the eastern Gotland Basin between 2006 and 2012 were analysed for organohalogen compounds of emerging concern (de Wit et al., 2020). Concentrations of new flame‐retardants (e.g. organophosphate esters ΣOPEs and chlorinated paraffins ΣCPs) in porpoises were similar to or exceeded those of polybrominated diphenyl ethers ΣPBDEs and hexabromocyclodecanes HBCDDs in the study. Little is known about the toxicity and potential combined effects of new and legacy compounds.

Concerning metal concentrations, there is growing concern about the health status of the harbour porpoise in the North Sea, Baltic Sea and adjacent areas (Ciesielski et al., 2006). Heavy metals accumulate throughout the lifespan of marine mammals, mainly via their diet (Das et al., 2004). Siebert et al. (1999) found significant associations between mercury levels, severity of pathological lesions and the nutritional state of harbour porpoises. Additionally, Desforges et al. (2021) have associated higher concentrations of total mercury with inhibition of the glutamate excitatory neurotransmitter in pilot whales (*Globicephala melas*), harbour porpoises, narwhals (*Monodon monoceros*), polar bears (*Ursus maritimus*) and ringed seals (*Pusa hispida*) from the Arctic. Harbour porpoises from the Kattegat/Skagerrak Seas and Norway present levels of mercury in the range of observed hepatic toxicity (Dietz et al., 2021). Baltic Proper harbour porpoises carry a significant mercury burden (Ciesielski et al., 2006; Szefer, Malinga, et al., 1995 , which has been associated with prevalence of parasitic infection and infectious diseases (Siebert et al., 1999). In a study on Baltic Sea harbour porpoises (*n* = 23), mercury concentration in the liver of one individual exceeded the negative effects threshold derived from dolphins (Roos, 2021). As well as mercury, the livers of two porpoises from Polish waters had markedly elevated levels of silver, indicating that they had been exposed to point sources of pollution (e.g. harbours or industrial plants) (Szefer et al.,^1995^ ). Additionally, increasing zinc levels were observed with deteriorating health conditions (emaciation and bronchopneumonia) in porpoises collected along the coasts of northern France, Belgium, Germany, Denmark, Iceland and Norway (Das et al., 2004), highlighting the interaction between contaminant exposure and health status. Despite the lack of data in the Baltic Proper, from other areas and mammal species, we have enough information on the negative consequences of mercury to conclude that action is required (Bennett et al. 2001; Kershaw et al. 2019).

Oil pollution is caused by major oil incidents, and from diffuse sources, such as leaks, illegal tank‐cleaning operations at sea, or discharges into rivers, which are then carried into the sea (Lavrova et al., 2014). The impact of oil pollution on harbour porpoises is unknown (Geraci, 1990). Oil can be swallowed or get into the respiration tract, and volatile components can be transferred into the blood (Geraci, 1990; Godard‐Codding & Collier 1990). In cetaceans, acute and chronic lung lesions as well as negative consequences for the immune system and reproductive success have been described (Barron, 2012; Lane et al., 2015; Matkin et al., 2008; Venn‐Watson et al., 2015).

##### Gaps in knowledge on environmental contaminants affecting conservation

The current contaminant levels in the Baltic Sea biota indicate that legacy and emerging contaminants remain a serious threat to the Baltic Proper harbour porpoise population, but scarcity of samples hamper comprehensive studies. The lack of samples is due to a combination of the small population size and a low willingness by fishermen to hand in bycaught harbour porpoises (Amundin et al., 2022). Stranding networks and post mortem investigations should be expanded so that any specimen stranded or handed in is investigated for cause of death, health parameters and contaminant levels.

Recent studies show that PCBs exposure continues to affect cetaceans including harbour porpoises in European waters (Jepson & Law, 2016; Desforges et al., 2018) with concentrations in blubber exceeding toxicity thresholds for mammals. PCB pollution is known to cause, for example, reproductive failure (Jepson et al., 2016) and increasing the risk of infectious disease (Hall et al., 2006) in odontocetes. However, the impact of exposure to PCB congeners on marine mammals is still largely unknown (Jepson et al., 2016) and it remains undetermined whether immunological changes are directly contaminant‐induced or a sequel of concurrent infectious diseases and poor health status, respectively (Lehnert et al., 2019). Studies from the southern part of the North Sea revealed that harbour porpoises with high PCBs concentrations died more often from an infectious disease and debilitations like emaciation than from an acute cause of death such as bycatch or predation (van den Heuvel‐Greve et al., 2021).

Little is known about many other chemical substances that exist in the marine environment that may affect the health status and reproductive success of harbour porpoises. For example, nothing is known about the long‐term effects of oil pollution on harbour porpoises. This especially relates to ecosystem effects of oil as well as chemical dispersants used in oil spills, which are relevant with respect to porpoise prey. Little is known about contaminants of emerging concern (CECs), including UV filters from sunscreen, agricultural and industrial chemicals, explosives and pharmaceuticals, their potential bioaccumulation, and effects on high trophic level predators. Wide scope target and suspect screening are promising means to detect and quantify CECs such as recently conducted in marine mammals of the Baltic Sea (de Wit et al., 2020). At present, there is not enough knowledge to assess CEC's impacts on Baltic Proper harbour porpoise. However, CECs presence in the environment and knowledge on their chemical characteristics and toxicity is sufficient to call for legislative action (Badry et al., 2022; Sonne et al., 2023).

### 3.2 Significant pressures on the population

#### 3.2.1 Prey depletion

##### Overview of the impact of prey depletion and implications for management

The harbour porpoise is one of the smallest cetacean species (Santos & Pierce, 2003). Relative to their body mass, harbour porpoises need large amounts of prey each day to sustain themselves and have been shown to forage almost continuously (Wisniewska et al., 2016). This makes them highly sensitive to prey depletion (Kastelein et al., 1997; Leopold & Meesters, 2015). Due to their relatively large body surface‐to‐volume ratio, they need to compensate for thermal energy loss in their cold water high‐latitude habitats to avoid hypothermia (Rojano‐Doñate et al., 2018). As an adaptation, the field metabolic rate (FMR) in this species is twice as high as that of similar‐sized terrestrial mammals (Rojano‐Doñate et al., 2018). It has been shown that the FMR is stable over seasonally fluctuating water temperatures, with heat loss managed via cyclical fluctuations in energy intake to build up the thermal insulation layer (blubber) for the cold season; reducing the cost of thermoregulation (Rojano‐Doñate et al., 2018). Further, females have a higher energy intake towards the end of pregnancy (March to July) and need a considerable amount of their yearly energy intake for reproduction and lactation, when blubber reserves are mobilised (Rojano‐Doñate et al., 2018; van den Heuvel‐Greve et al., 2021; Weijs et al., 2009).

Porpoises are intolerant of food shortage and can quickly die of starvation (Bjørge, 2003; Kanwisher & Sundnes, 1965; Kastelein et al., 1997; Koopman et al., 2002; Lockyer et al., 2003; Yasui & Gaskin, 1986). Prey quantity (number, size) and prey quality (energy content per prey item) are also important variables when considering the ability of an animal to maintain its energy budget and reproduce (IJsseldijk et al., 2021; Leopold & Meesters, 2015). To some extent, the depletion of one prey species can be compensated for by a shift to another if available in sufficient quality and quantity. A shift to small prey items eaten in large quantities may sustain porpoises as for small prey items they use a low‐energy foraging strategy (Rojano‐Doñate et al., 2024). However, this diet, which requires a large amount of time for feeding, makes the animals very susceptible to disturbances (Wisniewska et al., 2016). It is also known that reduced availability of high‐quality prey and shift to food of a lower energetic value can severely affect the individual viability (Leopold, 2015). Although it may not be the case for the diminished Baltic Proper population, findings from the Belt Sea population indicate that depletion of cod and herring as important prey, and a shift to small gobies, places individuals at their energetic limit (Rojano‐Doñate et al., 2018; Wisniewska et al., 2016), which can have population‐level consequences if occurring in the Baltic proper.

In the Baltic Sea, prey composition varies between areas, with the prey of harbour porpoises likely including pelagic schooling fish as well as demersal and benthic species (Aarefjord et al., 1995; Andreasen et al., 2017; Santos & Pierce, 2003; Sveegaard et al., 2012). Most dietary studies are from the Western Baltic, Belt Sea and Kattegat area where age, sex and seasonal differences were found in the diet of individuals (Andreasen et al., 2017; Sveegaard et al., 2012), and it is likely that the Baltic Proper population also has a diet that varies seasonally and throughout various life stages. The size of harbour porpoise prey in the Western Baltic Sea (data from 1980 to 2011) includes a wide range of lengths from approximately 2.5–63 cm, with 1‐ to 2‐year‐old cod and medium‐sized herring being the most common prey (Andreasen et al., 2017). Gobies (Gobiidae spp.) were also frequently consumed (25% of prey mass), especially by juveniles (Andreasen et al., 2017). Data from German waters of Mecklenburg‐Vorpommern (31 stomachs, 2013–2019) suggest that more than 90% of the diet, in terms of biomass, was small‐sized cod (mostly below 30 cm), followed by whiting (*Merlangius merlangus*) (Klemens, 2019). It should be noted that the porpoises in these studies have not been genetically assigned to a population. Based on the sampling locations, it is most likely that the majority of those analysed by Andreasen et al. (2017) and Klemens (2019) were animals from the Belt Sea population, and only a small proportion (if any) were from the Baltic Proper population. The shape of the skull of porpoises in Baltic Proper and Belt Sea populations is different, which is believed to indicate a morphological adaptation to demersal and benthic prey in the shallow Belt Seas and pelagic prey in the Baltic Proper (Galatius et al., 2012). The only dietary study available that likely targeted animals from the Baltic Proper is on individual's bycaught in the salmon driftnet fishery in 1960–1961 from Hanö Bight to the waters around Gotland (Lindroth, 1962). Based on stomach content analysis, the most common prey items of animals were sprat (*Sprattus sprattus*), transparent goby (*Aphia minuta*), herring and cod (Lindroth, 1962). It is unknown whether this still reflects the current diet of the Baltic Proper population.

In recent decades, considerable abiotic and biotic changes have taken place in the Baltic Sea (Reusch et al., 2018) and distorted the food web. Eutrophication related expansion of anoxic areas in the Baltic Proper (which now also affects shallow waters (e.g. spawning sites)) overexploitation of key fish species, in combination with climate change have caused ecosystem regime shifts in the Central Baltic Sea (Casini et al., 2008, 2012; Casini & Daniels, 2013; Eriksson et al., 2011; Möllmann et al., 2009), with implications for prey availability and diet composition of porpoises. By mainly targeting larger predatory fish for decades, commercial fisheries have caused a decrease in cod and herring, and indirectly, a rapid increase in the densities of some of their prey consisting of smaller mesopredators such as sprat, stickleback (*Gasterosteus aculeatus*) and gobies (Eriksson et al., 2011; Froese et al., 2022). These in turn feed on eggs and larvae of larger predatory fish during certain life history stages. Such ecosystem‐scale changes are further stabilised by continuing fisheries‐induced feedback loops in the food web (Andersson et al., 2023; Möllmann et al., 2009; Scotti et al., 2022).

Since the 1970s, most stocks of cod and herring in ICES subdivisions 25 to 32 in the Baltic Sea have decreased (ICES, 2022a, 2022b, 2023a, 2023b) mainly driven by fisheries (Bastardie et al., 2021; Froese et al., 2022; Möllmann et al., 2009). For the Eastern Baltic cod, spawning stock biomass is presently close to the lowest level observed since the 1950s (ICES, 2021, 2023a). The stock shows a regime shift from a high reproductive potential before the 1980s to currently a low potential (Voss & Quaas, 2022). Herring represents a low‐trophic level key species in the Baltic Sea food web because it transfers nutrients from zooplankton to higher trophic levels consisting of predatory fish and mammals (Scotti et al., 2022). Over the past years, the condition of Bothnian and central Baltic herring has deteriorated, and older and larger herring are less abundant (ICES, 2024). Their decline can lead to trophic cascades and contribute to a recent increase in three‐spined sticklebacks (Olin et al., 2022). Sticklebacks compete with herring for zooplankton in offshore areas and feed on herring eggs in coastal areas, which in turn can negatively affect recruitment success (ICES, 2022c; Olsson et al., 2019). The energy content of sprat can be very high (Pedersen & Hislop, 2005) and thus can be considered a high‐quality prey for porpoises. Their spawning stock biomass has fluctuated considerably due to a combination of top‐down and bottom‐up effects such as fishing pressure, recruitment, and natural mortality due to predator–prey relationships, especially with cod (Bastardie et al., 2021; ICES, 2022d). Sprat spawning stock biomass fluctuations still appear to be within safe biological limits in the Baltic Sea, according to biological benchmarks used in fisheries management (ICES, 2022d). However, modelling has shown that ‘business as usual’ for fisheries management would result in a high extinction risk of harbour porpoises in the western Baltic (Scotti et al., 2022). This modelling covered the range of the Belt Sea population, as well as a large part of the area of high importance for the Baltic Proper harbour porpoise along the Swedish east coast and in the southern Baltic Proper (see Figure 3 for areas of harbour porpoise importance). Alternatively, ecosystem‐based fisheries management would allow for the recovery of harbour porpoises and fisheries stocks, particularly cod and herring (Scotti et al., 2022).

Future predictions concerning changing hydrographic conditions of the Baltic Sea, and as a result, changing trophic cascades, suggest that several additional elements may be necessary for the proper assessment of maximum sustainable yield (MSY) for each stock. In addition to fishing pressure and species interactions, climate change, habitat loss and degradation, as well as eutrophication and growing areas of anoxia are predicted to be drivers of biomass, distribution, and condition of prey species of the Baltic Proper harbour porpoise (e.g. Bartolino et al., 2014; Bossier et al., 2021; Voss & Quaas, 2022) and thus need to be considered in determining MSY.

Even if the numbers of porpoises are currently only a small percentage of their historical abundance, the reduced quality and quantity of cod, herring and sprat, which are thought to be the main prey species for harbour porpoises in the Baltic Sea, may impede the recovery of the Baltic Proper harbour porpoises. The extraction of fish in the Baltic Sea overlaps with areas of high and medium importance for the Baltic Proper harbour porpoise (Figure 3). As a consequence, prey depletion is rated a significant pressure on the population.

##### Gaps in knowledge on prey depletion affecting conservation

The extent to which Baltic Proper harbour porpoises suffer from prey depletion is unclear, largely due to a lack of data. Measuring prey depletion requires current and baseline information on porpoise presence, prey preferences and prey availability (both in terms of quantity and quality), which are not available.

The current diet of Baltic Proper harbour porpoises is unknown with respect to prey species and size as well as seasonal, inter‐annual or individual variation. Whether or not dietary shifts are possible to compensate for resource limitation also needs to be analysed. This can include modelling energetic intake to help understand when limitations in prey availability begin to impact individuals and the population. Such models should also consider the low density of this severely depleted population, and the variety of cumulative pressures impacting the energetics and reproduction of the Baltic Proper harbour porpoise. Possible energetic consequences of distortions in the food web for Baltic Proper harbour porpoises have not been investigated yet. In particular, it is unknown to what extent mesopredators such as stickleback, sprat and gobies contribute to their diet, how the energetic requirements are met and how the body condition is affected by the current diet. This can have repercussions for the recovery potential of the population.

Existing stranding networks along Baltic Sea coasts have collected stomach contents from stranded and bycaught porpoises, which are available for hard part, metabarcoding or biochemical analyses of prey composition (Boyi et al., 2022). Expanding collections and establishing stranding networks would contribute to elucidating changes in diet in correlation with environmental change and between regions.

In order to have a full picture and understanding of the potential impact of prey depletion on the Baltic Proper population of harbour porpoise, sufficient monitoring of the changes in the distribution and quality of potential prey, concerning both commercial and non‐commercial fish species at spatial and temporal scales relevant to harbour porpoises would be needed. The main identified human pressures causing prey depletion can be cumulative, and the contribution of each pressure and their interaction is not completely understood. For example, the bioaccumulation of contaminants is mainly assembled via the diet in marine mammals, therefore diet composition will also influence the pollutant exposure in porpoises (Beineke et al., 2005; Fontaine et al., 2007). Generally, there is deficiency of information and data on how habitat loss and habitat degradation for prey species caused by eutrophication (Carstensen et al., 2014; Neuenfeldt et al., 2009) and other causes impact harbour porpoise distribution and foraging opportunities, for example, around hypoxic and anoxic areas in the Baltic Sea. However, overfishing is commonly accepted as one of the major threats causing prey depletion in the Baltic (Froese et al., 2022).

#### 3.2.2 Continuous noise

##### Overview of the impact of continuous noise and implications for management

In underwater habitats, hearing is a much more important sense than vision, as sound travels much further than light (Richardson et al., 1995). Harbour porpoises depend on echolocation to navigate and find prey (Koschinski et al., 2008; see Section 3.1.2), and there are indications from the closely related East Asian finless porpoise (*Neophocaena sunameri*) that passive listening may also be used for locating prey (Cheng et al., 2022). Harbour porpoises have a typical mammalian u‐shaped audiogram with the most sensitive hearing (defined as within 10 dB of maximum sensitivity) from 16 to 140 kHz (Kastelein et al., 2017b), sharply decreasing above, and with reasonable hearing at frequencies down to about 1 kHz. With respect to noise disturbance, harbour porpoises are especially sensitive to the mid‐ (1–10 kHz) and high‐frequency (10–140 kHz) part of the spectrum. At these frequencies, they elicit distinct behavioural responses, even at low levels (Dyndo et al., 2015; Wisniewska et al., 2018), suggesting that addressing noise at these frequencies is relevant to reduce disturbance and to contribute to species conservation.

Potential disturbance impacts include:

- Displacement, including avoidance behaviour (Wisniewska et al., 2018);
- Missed feeding or mating opportunities resulting from switching behavioural states, diving to the bottom suddenly, disruptions in foraging behaviour, reducing the rate of prey capture attempts, reducing echolocation activity or increasing fluking intensity (Bas et al., 2017; Dyndo et al., 2015; Frankish et al., 2023; Wisniewska et al., 2018);
- Masking of biologically significant sounds (Branstetter & Sills, 2022; Clark et al., 2009; Erbe et al., 2016; Hatch et al., 2012; Kinneging, 2023), and;
- Increased acute or chronic stress (Aguilar de Soto & Kight, 2016; Rolland et al., 2012; Wright et al., 2007).

The severity of the impact of noise‐induced disturbance on individual animals depends on how frequently they are exposed, as well as the intensity and duration of noise (see Section 3.1.2). The impact of frequent missed foraging opportunities can have energetic consequences for individuals (Rojano‐Doñate et al., 2024). If this affects the nutritional state, survival or reproductive success, negative population consequences are possible (National Research Council, 2005).

The amount of overlap between a signal and noise in time, space and frequency, but also other aspects such as the bandwidth or the amplitude modulation of the noise and further the angle between signal and masking noise determines the potential for masking (Richardson et al., 1995). Compared to marine mammals using low‐frequency signals for communication (such as seals and baleen whales), the echolocation and communication signals of harbour porpoises using frequencies much higher than most anthropogenic noise make them less vulnerable to masking (Richardson et al., 1995). Further, these signals are highly directional, and porpoises also have directional discrimination abilities at their echolocation frequency (Kastelein et al., 2005). At 1 and 10 kHz, the broadband component of noise from passing vessels at a distance of 1.2 km is estimated to cause hearing range reductions >20 dB (Hermannsen et al., 2014). This has the potential to mask other potentially biologically significant sounds at lower frequencies which are received by passive listening, for example, from prey, predators (e.g. grey seal (*Halichoerus grypus*)), fishing gear or other dangers (Erbe et al., 2016).

There are limited data on stress responses of porpoises to noise which are difficult to study in the wild (Elmegaard et al., 2023; Müller et al., 2013); however, there are some studies in captivity (Elmegaard et al., 2021; Teilmann et al., 2006). Some general principles can be concluded from studies on humans or other animal species including immediate (acute) release of stress hormones followed by changes in blood parameters (e.g. glucose). Long‐term effects are related to growth, behaviour, fertility and mortality and can be directly related to individual fitness and potentially have negative population impacts (Aguilar de Soto & Kight, 2016). Repeated or chronic stress in humans is linked to poor health conditions or effects in reproduction (Wright et al., 2011). Evidence of chronic stress in cetaceans related to ship noise exposure has been presented in North Atlantic right whales (Rolland et al., 2012).

Long exposures to high levels of continuous or intermittent sound can cause TTS or PTS in marine mammals (Finneran, 2015). In order to predict the risk of noise induced hearing loss, frequency weighted exposure thresholds have been developed (Southall et al., 2019). For low frequency continuous or intermittent noise there is strong support for thresholds with respect to hearing loss using the weighting function for very high‐frequency hearing specialists (Tougaard et al., 2022). However, between 10 and 20 kHz there is much variation between investigated individuals, and above 20 kHz there is a discrepancy between the predicted thresholds and recent experimental results which require further attention in future research (Tougaard et al., 2022).

The most commonly occurring source of continuous noise in the Baltic Sea is shipping, with major shipping lanes crossing areas of high and medium importance for Baltic Proper harbour porpoises (Figure 3). One of the main shipping lanes in the Baltic Sea intersects important porpoise habitat North of Rügen, North of Bornholm, Southeast of Öland and Southeast of Gotland (Figure 3). Shipping is often considered a low‐frequency noise, which is ubiquitous as background or ambient noise. The low frequency tones radiated from the propeller blades of large commercial vessels may not elicit behavioural responses in harbour porpoises, as the hearing sensitivity to those frequencies is low (Kastelein et al., 2017b). However, vessels also radiate broadband noise consisting of noise from engines, auxiliary machinery, and cavitation (Arveson & Vendittis, 2000; Wittekind, 2014). In the Kattegat and Great Belt, vessel noise substantially elevated the ambient noise in the entire recording band from 25 Hz to 160 kHz (Hermannsen et al., 2014). Cavitation is most pronounced if the propellers are poorly designed or the hull and propellers are not well maintained (Kendrick & Terweij, 2019). Above the vessel‐specific ‘cavitation inception speed’, cavitation noise is a function of speed. Cavitation noise is among the most disturbing noise sources from shipping, as it covers a broad range of frequencies including the range of best hearing for harbour porpoises (Arveson & Vendittis, 2000; Kastelein et al., 2017b; Wittekind, 2014). For most cargo vessels, reductions in speed are an effective and straight‐forward way of reducing cavitation noise emissions and the impact on marine mammals, a concept which could be adapted worldwide (Findlay et al., 2023; MacGillivray et al., 2019).

Recent research has shown that despite large changes in shipping noise and traffic in an area, passive acoustic monitoring of harbour porpoise detections in the Kattegat (likely the Belt Sea population) remained the same (Owen et al., 2024). No conclusions were able to be drawn on whether this continued use of preferred habitat was due to (1) a low impact of the observed level of change in vessel traffic and noise or (2) porpoises having no choice but to remain where prey are available, in an area with possibly increased disturbance and stress levels (Owen et al., 2024); and the population level impact of continuous noise remains unknown. Due to the low abundance of Baltic Proper harbour porpoises, their population is especially vulnerable against frequent disturbance.

In addition to commercial shipping, there are other sources of continuous noise that have the potential to cause strong behavioural responses from harbour porpoises. These include hydraulic or electric powered propulsion, for example, from dynamic positioning systems or thrusters that can create considerably strong tones with a range of harmonics extending to frequencies around 1 kHz (Richardson et al., 1995). Additionally, smaller recreational vessels or high‐speed vessels, frequently produce tonal sounds (and harmonics) at higher frequencies than most large commercial vessels, due to their generally higher revolutions per minute (rpm) (Dyndo et al., 2015; Wilson et al., 2022).

The MSFD Descriptor 11 (Energy and Noise) criterion D11C2 on ‘continuous low‐frequency sound’ states that ‘the spatial distribution, temporal extent and levels of anthropogenic continuous low‐frequency sound do not exceed levels that adversely affect populations of marine animals’. Similar to impulsive noise (Section 3.1.2), only population‐level effects are addressed by this criterion, and are often difficult to determine. According to the MSFD, Member States shall establish threshold values for spatial distribution, temporal extent and levels of anthropogenic continuous sound, taking into account regional or sub‐regional specificities. One of these specificities is the occurrence of the Critically Endangered Baltic Proper harbour porpoise population which, due to its status, requires a high level of precaution, especially in the light of the knowledge gaps outlined below. However, at the EU level, threshold values for continuous noise have only been developed for two frequency bands (64 and 125 Hz (Borsani et al., 2023)) where hearing in porpoises is poor (Kastelein et al., 2017b). Additionally, the HELCOM indicator on ‘Continuous low frequency anthropogenic sound’ also includes the 500 Hz decidecade band (HELCOM, 2023g). Therefore, the frequencies relevant to harbour porpoise disturbance are currently not assessed.

Another specificity in the Baltic Sea is that, due to the low salinity, the absorption of sound is lower than in oceanic waters (Richardson et al., 1995). Additionally, the stratified waters of the Baltic Sea sound channels could produce effects which locally increase sound propagation even further (Pihl et al., 2011; Sigray et al., 2016). These effects result in increased received levels of mid‐ and high‐frequency sounds, and greater impact ranges compared to oceanic environments. For this reason, more caution is needed when transferring research results from other marine areas to the Baltic Sea.

##### Gaps in knowledge on continuous noise affecting conservation

MSFD noise assessment calls for evaluation based on biologically adverse effects. Yet it is not clear what effects classify as biologically adverse, nor how much of the habitat can be affected for how long before it has population‐level impacts. Given that continuous noise is widespread, an understanding the impacts of such noise at the population level of Baltic Proper harbour porpoise is urgently needed. While there are a lack of data on the impact of continuous noise on the Baltic Proper harbour porpoise specifically, knowledge from other areas is relevant and useful, keeping in mind the likely larger impact distances in the Baltic Sea. The largest and most important knowledge gaps on the impact of continuous noise on harbour porpoises are the extent of energetic consequences of multiple disturbances, and what level of disturbance on individuals results in an impact on the population. The impact on individuals likely depends on the individual's current reproductive status, energetic requirements, nutritional state and the prey availability in terms of quantity and quality (Rojano‐Doñate et al., 2018, 2024). It is also likely that the extent of population‐level impact of disturbance varies throughout the year, between years and with function or importance of specific areas (Rojano‐Doñate et al., 2018). Loss of foraging opportunities could be particularly critical in autumn when porpoises build up their blubber needed for insulation. A continued decrease in energy intake may evoke a negative spiral of thermoregulatory loss due to a continuous mobilisation of energy from stores in the blubber (Rojano‐Doñate et al., 2018). This is why prey depletion is critical to be addressed in porpoise conservation because it limits the energetic compensation of such disturbances (see Section 3.2.1).

It can be concluded from the information above, that higher frequency bands than those currently assessed by the EU are very likely more relevant for harbour porpoises. It is currently unclear what area is affected by relevant frequency bands from continuous noise. Current MSFD monitoring also allows for the assessment of noise at higher frequencies bands and additional indicators should be developed at porpoise‐relevant frequencies, and also taking into account individual ship passages rather than ambient noise only. The HELCOM BLUES project (https://blues.helcom.fi/) has made considerable progress in noise mapping of commercial ships. However, noise mapping of recreational boats and other small‐speed craft is completely lacking (Hermannsen et al., 2019) and the impact of this noise source (alone, or cumulatively with other noise sources) is unknown.

It remains unclear whether avoidance behaviour by harbour porpoises is elicited by: (1) specific frequencies in the spectrum, (2) the whole spectrum as perceived by the animal or (3) the rate of change in received level, that is, the perceived movement of the noise source (Findlay et al., 2023; Wisniewska et al., 2018). Differences between vessels' source levels, frequency spectra and speed should be the focus of research on harbour porpoise behaviour in order to identify the most disturbing vessels. In the interim, it is realistic for managers to assume that the loudest vessels and also high‐speed vessels may be seen as the most disturbing and thus are of particular concern.

Many of the mechanisms of masking and masking release have been studied intensively in marine mammals under laboratory conditions (Branstetter & Sills, 2022). In the Baltic, effects need to be modelled and some level of precaution applied. For low‐frequency continuous noise, the ability to mask biologically relevant signals compromising prey–predator interactions and the ability to detect threats is of particular importance. It is not known if eavesdropping plays a role in avoiding dangers and locating prey and how this would be impacted by a reduction of the available bioacoustic space by continuous noise.

Further, since disturbance due to continuous noise acts cumulatively with other pressures such as prey depletion (Section 3.2.1) or contaminants (Section 3.1.3), methods to assess cumulative sub‐lethal effects, accounting for direct anthropogenic mortality from other pressures such as bycatch (Section 3.1.1) and impulsive noise (Section 3.1.2) need to be developed. Noise‐induced hearing loss by prolonged and repeated exposure within shipping lanes cannot be completely ruled out and requires attention at the species level. The possible physiological impact (cardiovascular and stress effects) of continuous noise exposure is understudied, preventing a meaningful assessment of these effects and the development of impact indicators.

#### 3.2.3 Infectious disease

##### Overview of the impact of infectious disease and implications for management

Infectious disease has the potential to cause high‐mortality rates in marine mammals in a short time‐frame (Sanderson & Alexander, 2020), which is of concern for small populations. The probability of mass mortalities caused by virus or bacterial pathogens increases with environmental change in marine systems and for marine mammals (Mazzariol, 2021; Sanderson & Alexander, 2020). Additionally, the growing evidence that susceptibility to infectious disease is enhanced by contaminant exposure of marine mammals (Busbee et al., 2020; Sonne et al., 2020) indicates that harbour porpoises in the Baltic Sea are more vulnerable to infectious pathogens. As a result, infectious disease is classed as a significant pressure on the Baltic Proper harbour porpoise population.

In a health assessment of by‐caught or stranded harbour porpoises from the Baltic coast of Latvia, Poland, Germany and Denmark between 1990 and 2015, 46%–100% of the animals were recognised as by‐caught individuals (Siebert et al., 2020). The respiratory tract was found to be the organ system with the highest number of pathological lesions.

Parasite prevalence, especially of pseudaliid nematodes is known to be high in harbour porpoises and infection levels are elevated in the Baltic Sea (Dzido et al., 2021; Lehnert et al., 2005; Wünschmann et al., 2001). In comparison, harbour porpoises from Greenland, Norway and Iceland had milder lungworm parasitism associated with a lower incidence of severe lesions, reflecting lower levels of environmental contaminants, and also differences in host populations and/or environmental circumstances in animals from German Baltic Sea waters (Wünschmann et al., 2001, Siebert et al., 2006., Lehnert et al., 2014) and resulting susceptibility to parasitic infections. In post‐mortem examinations on harbour porpoises collected in Swedish waters, six porpoises may have been individuals from the Baltic Proper population (Neimanis et al., 2022). Bacterial infections resulting in pneumonia were a frequent cause of death, and parasitic infections were common. In six animals diagnosed with lungworm infections, severe parasitic pneumonia was seen in five animals.

Cetacean morbilliviruses and papillomaviruses as well as *Brucella* spp. and *Toxoplasma gondii* are thought to induce high mortality rates, lower reproductive success and to increase the virulence of other diseases in cetaceans (Van Bressem et al., 1999, 2009). For this reason, population effects of infectious diseases are important to consider, especially in concert with environmental stressors like contaminant exposure, which seems to play a role in the emergence and pathogenicity of morbillivirus epidemics, lobomycosis/LLD, toxoplasmosis, poxvirus‐associated tattoo skin disease and infectious diseases of multifactorial aetiology in harbour porpoises (Van Bressem et al., 1999). A recent review on pathogens of marine mammals from the Baltic is available in Sonne et al. (2020). Severely diseased harbour porpoises showed a reduced proliferative capacity of peripheral blood lymphocytes together with diminished transcription of transforming growth factor‐b and tumour necrosis factor‐a, impaired function of peripheral blood leukocytes, indicating immune exhaustion and increased disease susceptibility, compared to healthy controls (Lehnert et al., 2019). Toxicological analyses performed in parallel revealed accumulation of PCBs, DDT and DDE in blood samples, potentially indicating increased disease susceptibility (Lehnert et al., 2019).

##### Knowledge gaps on infectious disease affecting conservation

Information on health status and infectious diseases of Baltic Proper harbour porpoises is scarce. Studies to investigate the disease factors and mortality aetiologies of harbour porpoises should continue through established stranding networks and systematic post‐mortem investigations along the Baltic Sea coasts (Siebert et al., 2020). Within HELCOM, as part of obligations to contribute data to marine mammal indicators reproductive status and blubber thickness (nutritional status), Member States need to record data on general health status of the animals. The number of investigated porpoises should be increased, for example, by an obligation to land bycaught porpoises. Additional research (including a meta‐analysis of necropsy data that were accumulated over time) would help to detect causes, seasonality, long‐term trends, frequency in different sex and age categories, and the environmental conditions that enhance the occurrence of infectious disease as well as emerging pathogens.

Changing environmental conditions, like salinity, temperature and pH, can influence developmental stages of trophically transmitted parasites in the food web and accelerate host–parasite interactions (Lakemeyer et al., 2020; Selbach et al., 2022). Anisakid nematodes and diphyllobothriid cestodes as well as zoonotic trematodes like *Pseudamphistomum truncatum* may undergo population dynamics due to global change (Mastick et al., 2024; Neimanis et al., 2016). Harbour porpoises at the top of the foodweb and as top predators are sentinels for ecosystem viability and porpoise population health can mirror the stability of the Baltic marine ecosystems and the effects of human activities on coastal and marine environments (Bossart, 2011; Peltier et al., 2013). Emerging infectious protozoans like Toxoplasma, Giardia, Cryprosporidium and bacteria, like Brucellosis, as well as virus spread across species boundaries (Stokholm et al., 2023; Thorsson et al., 2023). An outbreak can result from anthropogenic activity (Fayer et al., 2004) and seriously affect small coastal cetacean populations (Van Bressem et al., 2009). Infectious disease, contaminants and global change act in concert to create pressures and potentially cumulating threats to Baltic harbour porpoises. Monitoring health, diseases and causes of death of porpoises allows for identification of threats to this vulnerable population, to other animals and humans and their environment (Ijsseldijk et al. 2018), so that conservation action can be implemented timely.

### 3.3 Minor pressures on the population

There are several threats that result in minor or indirect pressures on the Baltic Proper harbour porpoise population, including climate change, habitat loss and degradation, litter (including micro‐sized litter and discarded fishing gear) and vessel strikes (details of the impact of these pressures on the Baltic Proper harbour porpoise are outlined in HELCOM, 2023h). These factors act cumulatively and indirectly impact the conservation of the Baltic Proper harbour porpoise.

Climate change can affect fish stocks and porpoise prey directly as warming decreases oxygen levels in seawater, or indirectly due to reduced recruitment and growth (e.g. by effects on the availability of food for larvae), or can impact the distributional range of species or their prey, for example, by changes in water temperature, salinity or ecological interactions (e.g. competition, also with invasive species) (MacKenzie et al., 2007). Additionally, a gradual but continued deterioration of fish habitat, especially in shallow coastal areas and estuaries is of particular concern for stocks of potential prey. Reasons for this could be coastal hypoxia, cascading effects of removal of predatory fish on plankton composition with increased blooms of mat‐forming filamentous algae and cyanobacteria (Casini et al., 2008; Eriksson et al., 2011; Reusch et al., 2018). Further, development and construction of infrastructure projects such as wind farms, pipelines or harbours, fish farms as well as bottom trawling, leading to physical alteration of fish habitats and often coincide with important areas for recruitment (Seitz et al., 2014), which can also have implications for prey availability for porpoises.

Marine litter is likely another minor pressure on the Baltic Proper harbour porpoise, with entanglement in disgarded fishing gear generally considered far more likely to cause of mortality than plastic ingestion (FAO, 2009). The main sources of marine litter include vessels (including fishing), offshore installations and land‐based sources (Cozar et al., 2014; Jambeck et al., 2015). Harbour porpoises stranded along the German North and Baltic Seas between 2014 and 2018 had a high frequency of occurrence of microplastics in the gastrointestinal tract and individuals in better nutritional status also had higher levels of microplastics (Philipp et al., 2021). Similarly, cetaceans that died due to infectious disease were shown to have a higher number of microplastic particles than those that died of other causes (Nelms et al., 2019). However, the question remains of how high microplastic ingestion and translocation affects health status or the uptake of PCBs and other POPs absorbed by ingested microplastic particles (Arthur & Baker, 2011; Philipp et al., 2021).

A vessel strike is defined as any impact between any part of a watercraft (most commonly bow or propeller) and a live marine animal (Peel et al., 2018). Injury indicative of vessel strikes such as blunt trauma, skull fracture or multiple lacerations is frequently reported from various parts of the world, including vessel strikes with harbour porpoises (IJsseldijk et al., 2022; Parsons & Jefferson, 2000; Sabin et al., 2005). In the Netherlands, blunt trauma, likely associated with vessel strikes was the cause of death in 4% of the 612 animals examined (IJsseldijk et al., 2022). Of these, 11 individuals had sharp‐edged mutilations likely from collision with vessel propellers, and 13 others presented possible evidence of a collision with a ship hull resulting in severe blunt force trauma (IJsseldijk et al., 2022). This indicates that while not well understood or documented, vessel strikes are likely to be an issue of conservation concern for harbour porpoises.

All of these minor pressures likely have a lower impact on the Baltic Proper harbour porpoise, and could be a lower priority for management action relative to the major and significant pressures described above.

## 4 DISCUSSION AND CONCLUSIONS

There is a lot of legislation aimed at protecting harbour porpoises in the EU region and sufficient knowledge to enforce this legislation now and prevent the extinction of this Critically Endangered marine mammal. From the review above, the following conclusions related to Baltic Proper harbour porpoises can be drawn:

- **There is evidence that bycatch is the main pressure and requires immediate and effective action.**

Bycatch mitigation can work and allow depleted porpoise populations to recover. For example, in 2020, all commercial fishing was banned in the Yangtze River in China (Mei et al., 2020). Since then, over the last 5 years, the Yangtze finless porpoise (*Neophocaena asiaorientalis asiaorientalis*) has increased 23% in abundance, after decades of decline (WWF China, 2023). Additionally, in California in the United States of America, an area that also has high PCB concentrations and levels of shipping traffic, regulatory action on the use of pingers, depth of fishing and large time‐area closures to fishing activities (Barlow & Cameron, 2003; Moore et al., 2009), followed by a permanent prohibition in set gillnet fishing in waters shallower than 110 m allowed the recovery of the Morro Bay harbour porpoise population (Forney et al., 2020).

It is evident that the Baltic Proper harbour porpoise population cannot withstand current bycatch levels, even on the basis of incomplete data. Based on available life history data, bycatch of this population must be zero to allow recovery. Given the difficulties associated with robustly assessing the level of bycatch, the only way to ensure zero bycatch of this Critically Endangered population is the implementation of effective bycatch mitigation measures in the whole distribution range. Such mitigation measures include static net closures, changing of gear type, reductions in effort, gear modifications (such as pingers), or limitations on soak time. A strict bycatch reporting obligation and landing of porpoises for the whole area may help close the data gaps outlined here. Improved data on fishing effort for the relevant fishing types and vessel sizes will help determine the areas of high risk for bycatch, which could change over time.

b **Avoid unprotected underwater explosions.**

Explosions of UXO have been linked to blast injuries and the death of harbour porpoises in the Baltic Sea. In small populations of long‐lived late reproducing species, the direct mortality associated with explosions (e.g. during UXO clearance) affects the population. Even in larger harbour porpoise populations, a population impact of regular UXO clearing activities has been shown (von Benda‐Beckmann et al., 2015). Explosions are the loudest point source emitters of impulsive noise. They are among the most commonly occurring events of impulsive noise sources in the Baltic Sea (HELCOM, 2023e). Therefore, UXO explosions in situ should be avoided and munitions recovered wherever possible. If this is not possible due to safety reasons, relevant noise/blast mitigation measures including, for example, bubble curtains should be implemented.

c **There is a need to regulate and limit the use of emerging noise sources such as seal scarers and acoustic antifouling devices.**

Licencing and/or limiting seal scarers and acoustic antifouling devices will help to minimise their impacts. The HELCOM Regional Action Plan for Underwater Noise (HELCOM, 2021b) proposes to develop and agree on common guidelines and regulations of the design and use of deterrent devices. Due to their large impact area, widespread use of seal scarers (such as in fisheries and aquaculture) should be avoided. There is also a need to investigate and regulate acoustic antifouling devices. A regulation must be introduced at an early stage before these devices become ubiquitous in the Baltic Sea.

d **There is a general need to avoid contaminants and waste entering the marine environment as they negatively influence harbour porpoise health, reproduction and survival.**

Emissions of hazardous substances especially persistent, bioaccumulative and toxic (so‐called PBT) substances to the marine environment should be further reduced. Although there are only a few samples available to study contaminants in Baltic Proper harbour porpoise tissues, high contaminant concentrations are known to affect the health status, increasing the risk of reproductive failure and infectious disease. This threat is of particular importance in the Baltic Sea where higher concentrations of legacy and emerging contaminants (e.g. PFAS, mercury and PCBs) in biota are observed compared to other parts of the world. Actions of the BSAP relating to hazardous substances should be ambitiously implemented. Even in the event of reduced pollutant sources, contaminant levels in the environment persist and accumulate along trophic levels, resulting in long lasting exposure and effects. Taking this knowledge into account, the prevention of new contaminant compounds (CECs) entering the food web should be of utmost importance.

e **The Baltic Proper food web is distorted which can impact prey quality and quantity for the Baltic Proper harbour porpoise. It is important to introduce ecosystem‐based sustainable management of fisheries, aquaculture and agriculture, in order to restore and maintain a functioning food web and a healthy Baltic Sea.**

The availability and quality of porpoise prey are affected by fisheries, eutrophication, climate change and other factors. These multiple pressures on the food web require a multi‐sectoral approach aiming at the recovery of fish stocks and natural trophic interactions to increase the resilience of the Baltic ecosystem. Trends in the removal of predatory fish, declining fish stocks, reduced mean fish size and reduced recruitment and growth need to be reversed. When determining the total allowable catch (TAC), ecosystem‐based management needs to be implemented in fisheries, taking into account possible low recruitment of fish species. When determining MSY for each fish stock, trophic interactions between fish species and thus recovery plans for multiple fish species, including non‐commercially exploited fish species, need to be considered. Better understanding of trophic cascade effects need to be a focal area of fisheries research in the Baltic Sea.

f **Conservation action is needed in all sectors across all anthropogenic activities.**

Measures that have a direct effect on the population by directly reducing anthropogenic mortality are most effective. Measures limiting bycatch and fatalities caused by explosions fall in this category. Other measures act indirectly by improving the nutritional state or reproductive success of individuals. All actions aiming at restoring fish stocks (including reductions in eutrophication), lowering pollution or reducing disturbance fall under this category. Most actions aimed at significantly reducing the identified threats require far‐sightedness and patience, as these can be determined to be effective only after significant time periods due to the low reproductive rate and the small size of the porpoise population.

g **Cumulative pressures need to be taken into account. Disturbance (e.g. by underwater noise) is worse when prey is depleted and missed feeding opportunities cannot be fully compensated for.**

Harbour porpoises are vulnerable to disturbance due to their high metabolic rate and the need to feed constantly. Consequently, the prey species, size and energy content matter as it determines how many missed feeding opportunities can be tolerated. Research on prey composition and monitoring of nutritional status are a first step to better understand the energetic consequences of disturbance for individuals and possible impact on the population. It is likely that autumn when blubber is deposited to prepare for the winter, and spring when more energy is required for pregnancy and lactation, are especially sensitive periods. With fertility and reproductive success impacted by environmental contaminants, all other threats need to be minimised to allow the population to recover.

h **There is an urgent need for an updated abundance estimate and new information on the distribution of the population in order to best position protective measures and define important areas.**

Currently there is only a single abundance estimate for the Baltic Proper population of harbour porpoises, which is over a decade old. This makes it difficult to assess the effect of any conservation measures that are introduced and to determine the current status of the population. Additionally, the only maps of population distribution across its range are over a decade old. While the available information is suitable to inform management, updated information ensures that management actions are targeted to the most important areas for the population and will have the strongest result. Multinational collaborative studies to assess abundance and distribution, such as the planned SAMBAH II project and initiation of harmonised monitoring programmes for harbour porpoises across the Baltic Sea region are urgently needed.

i **A common database including the cause of death, health status, contaminant load, diet and population assignment of each animal investigated would help in quantifying the population‐level impact of each activity.**

Many knowledge gaps can be addressed by increasing the stranding networks and post mortem investigations around the Baltic Sea in order to improve the availability of data from all stranded and bycaught animals. Such programmes would allow for better monitoring of the population status and contribute a wealth of information, that is also required by HELCOM to feed into core indicators for reproductive status and blubber thickness, as well as a health indicator under development (and possibly a pollutant indicator that will be harmonised with the OSPAR indicator and adapted for the Baltic Sea region). Post mortem investigations are useful to assess changes in health, detect infectious pathogens of zoonotic importance, causes of death, demographic parameters, diet and nutritional status. Additionally, samples can support genetic analyses to determine changes in population abundance over time and designate individuals to the Baltic Proper population. Databases currently available or under development should be coordinated for data sharing.

j **There are still a number of knowledge gaps, but information necessary for protection is known or can be derived from other porpoise populations.**

In conservation, the precautionary principle should be used, especially when dealing with Critically Endangered populations, as it allows for the implementation of measures immediately, even when knowledge gaps about the specific population are present. The available knowledge, in some cases gained either from other harbour porpoise populations, or at an individual level, demonstrates the vulnerability of harbour porpoise populations towards all of the pressures discussed. While understanding the population‐level impacts of pressures is important, for extremely small populations such as the Baltic Proper harbour porpoise population, any impact on an individual will have a population level impact. Additionally, much of the required population‐specific knowledge may be impossible to obtain until after the population recovers, meaning that in the majority of cases reliance on data from neighbouring populations is the only option to aid conservation. In some cases, it may be possible that new methods, such as acoustic analyses to detect the presence of calves (e.g. see Delgado‐García, 2016), can assist with filling in some of the many knowledge gaps in this population. However, we already know enough to act now.

k **Extinction of this population is a choice, meaning that decision‐makers have it in their hands. It is known what caused the decline of this population and what measures are needed. The instruments for effective protection are at hand.**

The extinction risk increases with decreasing population size. There is no time to waste. Recovery of this population is possible. Adaptive management is needed, where fast and proactive decisions to protect the population are put in place now.

## AUTHOR CONTRIBUTIONS

**Sven Koschinski:** Conceptualization (equal); funding acquisition (equal); investigation (equal); writing – original draft (equal); writing – review and editing (equal). **Kylie Owen:** Conceptualization (equal); funding acquisition (equal); investigation (equal); writing – original draft (equal); writing – review and editing (equal). **Kristina Lehnert:** Conceptualization (equal); funding aquisition (equal); investigation (equal); writing – original draft (equal); writing – review and editing (equal). **Katarzyna Kamińska:** Conceptualization (equal); investigation (equal); writing – original draft (equal); writing – review and editing (equal).

## CONFLICT OF INTEREST STATEMENT

The authors declare no competing interested.

## Data Availability

Data sharing not applicable to this article as no datasets were generated or analysed during the current study.
