# Correction to “Current Species Protection Does Not Serve Its Porpoise—Knowledge Gaps on the Impact of Pressures on the Critically Endangered Baltic Proper Harbour Porpoise Population, and Future Recommendations for Its Protection”

**DOI:** 10.1002/ece3.70445

**Published:** 2024-10-11

Koschinski, S., K. Owen, K. Lehnert, and K. Kamińska. 2024. “Current Species Protection Does Not Serve Its Porpoise—Knowledge Gaps on the Impact of Pressures on the Critically Endangered Baltic Proper Harbour Porpoise Population, and Future Recommendations for Its Protection.” *Ecology and Evolution* 14, e70156.

Proposed management borders were not correctly shown in Figures 1 and 2.

**FIGURE 1:**
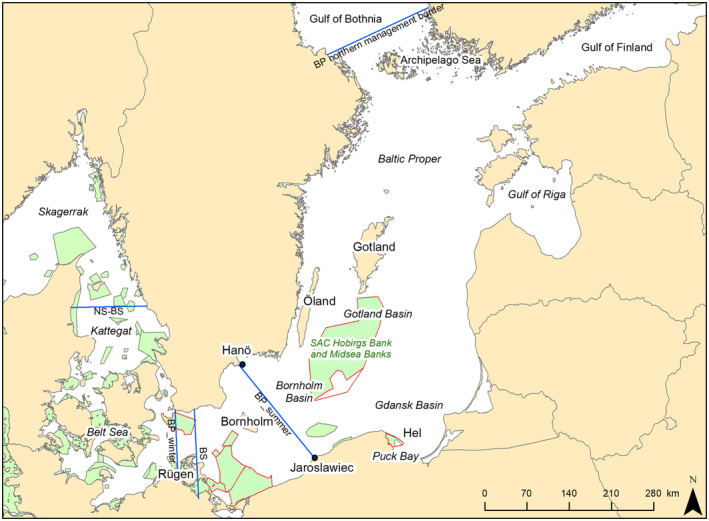
Map of the management borders for all three harbour porpoise (*Phocoena phocoena*) populations in the Baltic Sea region (North Sea population [NS], Belt Sea population [BS] and the Baltic Proper population [BP]) are shown. NS‐BS based on Sveegaard et al. (2015); BS based on Sveegaard et al. (2015, 2022); BP_winter based on ICES (2020b), Sveegaard et al. (2022); BP_summer based on Carlén et al. (2018), Sveegaard et al. (2022); BP northern management border based on ICES (2020b). The Natura 2000 sites where harbour porpoises are listed are shown in green. Sites outlined in red are those to which seasonal or year‐round closures for fisheries apply, or obligation to use pingers on static nets was implemented.

**FIGURE 2:**
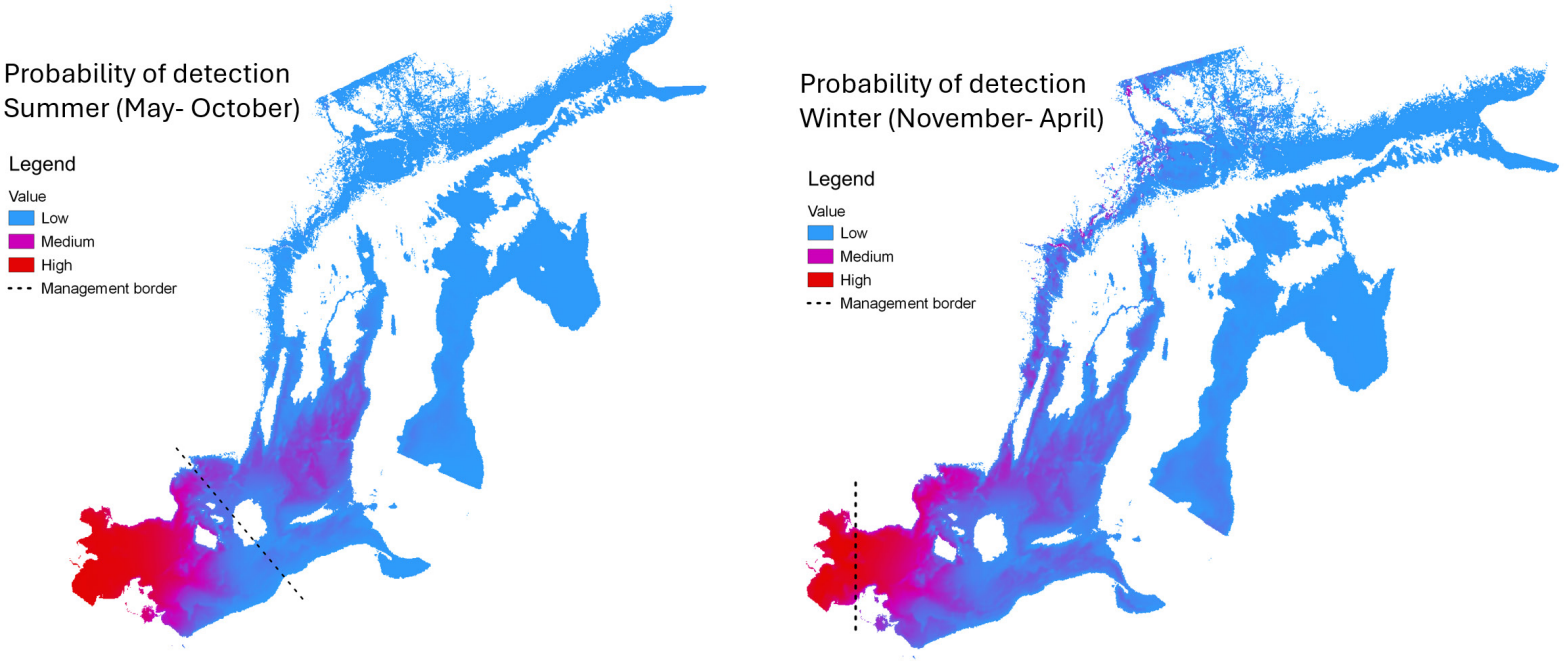
Summer (May–October, left) and winter (November–April, right) detection probabilities of the Baltic Proper harbour porpoise (*Phocoena phocoena*), as collected during the SAMBAH project (Carlén et al. 2018).

We apologise for this error.

